# A Comparative Analysis of Genetic Ancestry and Admixture in the Colombian Populations of Chocó and Medellín

**DOI:** 10.1534/g3.117.1118

**Published:** 2017-08-28

**Authors:** Andrew B. Conley, Lavanya Rishishwar, Emily T. Norris, Augusto Valderrama-Aguirre, Leonardo Mariño-Ramírez, Miguel A. Medina-Rivas, I. King Jordan

**Affiliations:** * Applied Bioinformatics Laboratory, Atlanta, Georgia; † PanAmerican Bioinformatics Institute, Cali, Valle del Cauca, Colombia; ‡ School of Biological Sciences, Georgia Institute of Technology, Atlanta, Georgia 30332; § Biomedical Research Institute, Universidad Libre, Cali, Valle del Cauca, Colombia; ** National Center for Biotechnology Information, National Library of Medicine, National Institutes of Health, Bethesda, Maryland 20894; †† Centro de Investigación en Biodiversidad y Hábitat, Universidad Tecnológica del Chocó, Quibdó, Chocó, Colombia 270002

**Keywords:** Population Genetics, Admixture, Genetic Ancestry, Comparative Genomics, Human Genomics, Afro-Colombian, Afro-Latino

## Abstract

At least 20% of Colombians identify as having African ancestry, yielding the second largest population of Afro-descendants in Latin America. To date, there have been relatively few studies focused on the genetic ancestry of Afro-Latino populations. We report a comparative analysis of the genetic ancestry of Chocó, a state located on Colombia’s Pacific coast with a population that is >80% Afro-Colombian. We compared genome-wide patterns of genetic ancestry and admixture for Chocó to six other admixed American populations, with an emphasis on a Mestizo population from the nearby Colombian city of Medellín. One hundred sample donors from Chocó were genotyped across 610,545 genomic sites and compared with 94 publicly available whole genome sequences from Medellín. At the continental level, Chocó shows mostly African genetic ancestry (76%) with a nearly even split between European (13%) and Native American (11%) fractions, whereas Medellín has primarily European ancestry (75%), followed by Native American (18%) and African (7%). Sample donors from Chocó self-identify as having more African ancestry, and conversely less European and Native American ancestry, than can be genetically inferred, as opposed to what we previously found for Medellín, where individuals tend to overestimate levels of European ancestry. We developed a novel approach for subcontinental ancestry assignment, which allowed us to characterize subcontinental source populations for each of the three distinct continental ancestry fractions separately. Despite the clear differences between Chocó and Medellín at the level of continental ancestry, the two populations show overall patterns of subcontinental ancestry that are highly similar. Their African subcontinental ancestries are only slightly different, with Chocó showing more exclusive shared ancestry with the modern Yoruba (Nigerian) population, and Medellín having relatively more shared ancestry with West African populations in Sierra Leone and Gambia. Both populations show very similar Spanish ancestry within Europe and virtually identical patterns of Native American ancestry, with main contributions from the Embera and Waunana tribes. When the three subcontinental ancestry components are considered jointly, the populations of Chocó and Medellín are shown to be most closely related, to the exclusion of the other admixed American populations that we analyzed. We consider the implications of the existence of shared subcontinental ancestries for Colombian populations that appear, at first glance, to be clearly distinct with respect to competing notions of national identity that emphasize ethnic mixing (*mestizaje*) *vs.* group-specific identities (multiculturalism).

Like many countries in Latin America, Colombia is home to a diverse, multi-ethnic society (Hernández Romero 2005). There are a number of distinct population groups in the country, each of which is made up of individuals with varying degrees of African, European, and/or Native American ancestry (CIA 2014), and the modern population of Colombia has been shaped by high levels of genetic admixture between these three ancestral source populations (Rishishwar *et al.* 2015b; Wang *et al.* 2008). In fact, recent studies of genetic ancestry indicate that Colombia has among the highest levels of three-way admixture, in terms of substantial ancestry contributions from all three source population groups, seen for any Latin American country (Bryc *et al.* 2010b; Homburger *et al.* 2015; Ruiz-Linares *et al.* 2014). There is also extensive geographic population structure in Colombia, with populations from different regions in the country having very distinct ancestry profiles (Hernández Romero 2005).

To date, most studies on human genetic ancestry in Colombia, and throughout Latin America for that matter, have focused on Native American populations or Mestizo populations that have a mix of European and Native American ancestry (Cordoba *et al.* 2012; Carvajal-Carmona *et al.* 2000, 2003; Bedoya *et al.* 2006; Wang *et al.* 2008; Bryc *et al.* 2010b). However, Colombia is also home to a large population of Afro-descendants. The size of the Afro-Colombian population was estimated to be ∼5 million as of the 2005 census (Hernández Romero 2005), making it the second largest population of its kind for any country in Latin America, after Brazil. Despite the substantial presence of Afro-descendants in the country, there have been relatively few studies on the genetic ancestry of Afro-Colombian populations (Ansari-Pour *et al.* 2016; Medina-Rivas *et al.* 2016; Rishishwar *et al.* 2015a). A more robust understanding of Latin American genetic ancestry, particularly for Colombia, will require additional studies of Afro-Latino populations.

The ChocoGen research project was initiated to facilitate genetic studies of a predominantly Afro-Colombian population (http://www.chocogen.com/). ChocoGen is a collaboration between the Universidad Tecnológica del Chocó (UTCH) and a number of partner institutions in Colombia and the United States. Project investigators are working to characterize the genetic ancestry of the Colombian administrative department (*i.e.*, state) of Chocó and to develop local capacity for research and education in human genomics. The state of Chocó, which borders Panamá to the north and stretches south along Colombia’s Pacific coast, has a population that is >80% Afro-Colombian (Hernández Romero 2005). Our first study of the population of Chocó provided a high-level view of its genetic ancestry, underscoring the African heritage of the region along with its relatively high levels of admixture and genetic diversity (Medina-Rivas *et al.* 2016). The population of Chocó was found to have similar overall levels of African ancestry, but far more Native American ancestry, compared with African American and African Caribbean populations characterized as part of the 1000 Genomes Project (1KGP) (1000 Genomes Project Consortium *et al.* 2010, 2012, 2015). Accordingly, Chocó shows higher levels of three-way African-European-Native American admixture than these other New World African populations. The population of Chocó also has higher levels of overall genetic diversity compared with both ancestral source populations and other admixed Latin American populations. Consistent with results seen for Mestizo populations in Latin America, Chocó shows a highly asymmetric, sex-specific pattern for the non-African component of its ancestry, with predominantly Native American ancestry along the female lineage and European ancestry along the male lineage.

The Afro-Colombian makeup of the population of Chocó, along with its distinctive conditions of isolation and marginalization, can be traced to the historical and economic development of the region (Sharp 1976). Africans first arrived in Colombia through the notorious slave port city of Cartagena, situated on the country’s Caribbean coast (De Friedemann 1993). Cartagena was the main port of entry for African slaves destined for the Spanish colonies, and it has been estimated that as many as one million Africans may have been forcibly brought to Cartagena over the three centuries of the transatlantic slave trade. An analysis of transatlantic slave voyage records from 1533 to 1810 documented the arrival of ∼550,000 Africans in Cartagena across three distinct eras of forced migration (Maya Restrepo 2005; Rodriguez 2008) (Supplemental Material, Figure S1 in File S1). It is thought that Africans were first brought to Chocó as early as the 1670s by Spaniards in search of gold (Mosquera *et al.* 2002; Mosquera 2014). One of the first narrative accounts of the African presence in Chocó dates to 1690 when a *cuadrilla*, a working group of slaves numbering from ∼30–100 individuals, was sent from Popayán to work in the region’s gold mines (Colmenares 1979). The population of African slaves in Chocó had grown to ∼2000 by 1724, and in 1778 there were ∼5800 slaves and ∼3300 free blacks living in Chocó (Wade 1990). Afro-Colombians were already a substantial majority in the region by that time, making up 61% of the population compared with a 37% indigenous component and only a 2% white component.

The colonial economy of Chocó was almost exclusively extractive in nature, wherein the region’s gold riches were destined solely for exportation, and there was little or no effort by the white ruling and managerial classes to establish a lasting presence in the region (Sharp 1976; Wade 1990). Although independence and emancipation brought some changes to Chocó, culminating with the political ascendancy of the Afro-Colombian population in the middle of the 20th century, the structural inequalities and fundamental nature of the economy based on exploitation and extraction remained largely unchanged. Meanwhile, political empowerment of Afro-Colombians in Chocó, along with their increasing access to its elite educational institutions, precipitated an exodus of many of the whites that had lived in the urban centers. The Colombian government for its part, although more than willing to benefit from the region’s exported wealth, consistently neglected to provide basic services to Chocó, such as healthcare, drinking water, and physical infrastructure. In addition, Chocó has always been known as a terribly inhospitable jungle region, with extremes of heat, humidity, and rain along with a debilitating burden of infectious disease, primarily malaria. The hostile climate and topology of Chocó simultaneously served to discourage settlement by whites while providing a refuge for escaped slaves, and a relatively large population of free blacks, who wished to live beyond the reach of colonial control. Because of these interlaced economic, social, and physical conditions, the population of Chocó has remained largely Afro-Colombian, as well as poor and marginalized, throughout the colonial and republican periods of Colombia’s history until the present day.

The aim of this study was to provide a detailed picture of the genetic ancestry and admixture patterns for an Afro-Colombian population and to compare its ancestry to other admixed American populations. Part of this aim includes an effort to provide a more inclusive and accurate sense of what it means to be Colombian (Latino) from a genetic perspective. We were particularly interested in comparing the genetic ancestry and patterns of admixture seen for the Afro-Colombian population of Chocó and a Mestizo population from Medellín in the nearby state of Antioquia. We consider the results of our comparative ancestry and admixture analyses in the context of two competing notions regarding ethnic and cultural identity in Latin America: *mestizaje* *vs.* multiculturalism (Chavez and Zambrano 2006). *Mestizaje* entails the intentional blending of ethnic groups to create a single, coherent national identity, whereas multiculturalism emphasizes a more explicitly pluralistic worldview, with distinct identities realized for numerous population groups within a country.

## Materials and Methods

### Chocó sample donors and genotyping

The genotype data used for this study are taken from the 100 sample donors from the population of Chocó, who were recruited from UTCH, as previously described (Medina-Rivas *et al.* 2016). Donors were selected in an effort to include representative samples of different geographic regions of Chocó: Atrato, Baudó, Costa Norte, Costa Pacífica, and San Juan (Figure S2 in File S1 and Table 1), and an approximately equal representation of males and females was included. Donors contributed DNA using a noninvasive saliva sampling method. All donors signed informed consent documents indicating their understanding of the potential risks of the project, along with how their data would be handled and how their identity would be protected. Collection, genotyping, and comparative analyses of human DNA samples were conducted with the approval of the Ethics Committee of UTCH. Donor DNA samples were genotyped using the Illumina HumanOmniExpress-24 single-nucleotide polymorphism (SNP) array. The KING program (Thornton *et al.* 2012) was used to test for kinship among the sample donors from Chocó, resulting in the identification of three sets of related individuals. All but one representative individual was removed for each set of related individuals, yielding a final set of 94 unrelated individual genotypes.

**Table 1:** Human populations analyzed in this study

	Dataset*^a^*	Full Description	Short	*n*		Dataset	Full Description	Short	*n*
Colombia (*n* = 228)	Medina-Rivas *et al.* (2016)	Chocoano in Quibdó, Colombia	Chocó	94	Mesoamerica (*n* = 306)	1KGP	Mexican Ancestry in Los Angeles, California	Mexico	64
	1KGP	Colombian in Medellin, Colombia	Medellín	94		1KGP	Puerto Rican in Puerto Rico	Puerto Rico	104
	Reich *et al.* (2012)	Native American tribes	Kogi	4		HGDP	Native American tribes	Pima	14
	Reich *et al.* (2012)		Waunana	3		Reich *et al.* (2012)		Tepehuano	25
	Reich *et al.* (2012)		Embera	5		Reich *et al.* (2012)		Mixtec	5
	Reich *et al.* (2012)		Guahibo	6		HGDP		Maya	21
	Reich *et al.* (2012)		Piapoco	7		Reich *et al.* (2012)		Mixe	17
	Reich *et al.* (2012)		Inga	9		Reich *et al.* (2012)		Zapotec	43
	Reich *et al.* (2012)		Ticuna	6		Reich *et al.* (2012)		Kaqchikel	13
South America (*n* = 187)	1KGP	Peruvian in Lima, Peru	Peru	85	Europe (*n* = 471)	1KGP	Finnish in Finland	Finnish	99
	Reich *et al.* (2012)	Native American tribes	Aymara	23		1KGP	British in England and Scotland	British	90
	Reich *et al.* (2012)		Guarani	6		1KGP	Iberian populations in Spain	Spanish	107
	Reich *et al.* (2012)		Quechua	40		1KGP	Toscani in Italy	Tuscan	107
	HGDP		Surui	8		HGDP		Russian	25
	Reich *et al.* (2012)		Wayuu	11		HGDP		Orcadian	15
Africa (*n* = 547)	1KGP	Esan in Nigeria	Esan	99	North American (*n* = 186)	HGDP		French	28
	1KGP	Gambian in Western Division, The Gambia	Gambian	113		1KGP	African Ancestry in Southwest United States	African American	61
	1KGP	Luhya in Webuye, Kenya	Luhya	99		1KGP	African Caribbean in African Caribbean	African Caribbean	96
	1KGP	Mende in Sierra Leone	Mende	85		Reich *et al.* (2012)	Native American tribes	Chipewyan	15
	1KGP	Yoruba in Ibadan, Nigeria	Yoruba	108		Reich *et al.* (2012)		Cree	4
	HGDP		Mandenka	22		Reich *et al.* (2012)		Ojibwa	5
	HGDP		Yoruba	21		Reich *et al.* (2012)		Algonquin	5

Populations are organized into continental groups, and the number of individuals in each population is shown.

a 1KGP, 1000 Genomes Project (phase 3 data release); HGDP, Human Genome Diversity Project Reich *et al.* (2012) refers to a recent study of Native American genetic ancestry (2012).

### Comparative genomic data

The genotypes of sample donors from Chocó were compared with whole genome sequence data from the 1KGP (1000 Genomes Project Consortium *et al.* 2010, 2015) and genotype data from the Human Genome Diversity Project (Cann *et al.* 2002; Jakobsson *et al.* 2008; Li *et al.* 2008) (Table 1). A collection of Native American genotypes from 21 populations, characterized using several SNP array platforms as previously described, was taken from a recent study on Native American population history (Reich *et al.* 2012). These Native American genotype data were accessed according to the terms of a data use agreement from the Universidad de Antioquia. Genotypes from the Chocó sample donors were merged with the comparative genomic data sources using PLINK version 1.9 (Chang *et al.* 2015), keeping only those sites common to all datasets and correcting SNP strand orientations as needed. The final merged dataset includes 239,989 SNPs across 2404 individuals.

The merged genotype dataset was phased using ShapeIT version 2.r837 (Delaneau *et al.* 2013). SNPs that interfered with the ShapeIT2 phasing process were excluded from subsequent analyses. ShapeIT2 was run without reference haplotypes, and all individuals were phased at the same time. Individual chromosomes were phased separately, and the X chromosome was phased with the additional “-X” flag. PLINK was used to prune linked SNPs from the phased genotype dataset, using the indep-pairwise functionality and arguments 50 10 0.1, keeping 58,898 unlinked SNPs for subsequent analysis with the program ADMIXTURE (Alexander *et al.* 2009).

### Ancestry inference

Continental ancestry fractions for the admixed American populations were inferred from the pruned SNP set using ADMIXTURE with *K* = 3 ancestral populations, using the African, European, and Native American reference populations shown in Table 1. The RFMix program (Maples *et al.* 2013), version 1.5.4, was used for both local ancestry inference and to obtain a corresponding set of rephased genotypes. RFMix was run in the PopPhased mode with a minimum node size of five, using 12 generations and the “use-reference-panels-in-EM” for two rounds of EM, generating local ancestry inference for both the reference and admixed populations. Continental African, European, and Native American populations were used as reference populations. Contiguous regions of ancestral assignment, “ancestry tracts,” were created where RFMix ancestral certainty was at least 99%.

Subcontinental ancestry fractions were inferred using ADMIXTURE with a novel approach we developed that uses output from the RFMix program. The idea behind this hybrid approach is to constrain the subcontinental ancestry inference to the regions of the genome(s) that correspond to a single continental ancestry at a time. This is done by running RFMix first, and then using the output for ADMIXTURE. The genomic regions that correspond to each individual continental ancestry fraction (African, European, and Native American) were characterized using RFMix as described above. Then, for each continental ancestry, the genomic regions from the other two continental ancestries were treated as missing. Individuals with <10% genotyping rate after masking were left out of the analysis. The resulting continental ancestry–specific genotypes are used as input for three separate runs of ADMIXTURE analysis. ADMIXTURE was run with 100 replicates for all three continental ancestries, using *K* = 3 (European and African) and *K* = 14 (Native American).

We performed an analogous hybrid approach for subcontinental ancestry characterization that combines ChromoPainter2 analysis (Lawson *et al.* 2012) with RFMix output. In this case, ChromoPainter2 is run first, and then the RFMix local ancestry calls are used to refine the results. ChromoPainter2 characterizes local ancestry by comparing the genotypes of the admixed individuals to genotypes from continental ancestry reference populations. For our hybrid approach, ChromoPainter2 was run first to characterize the best matches between the haplotypes of admixed individuals and haplotypes from the three continental ancestry reference populations. This was done in three separate runs for each distinct continental ancestry fraction: African, European, and Native American. For each continental ancestry, the resulting genome-wide distributions of local ancestry (*i.e.*, matches to the reference genome haplotypes) were filtered using RFMix. The RFMix filtering entailed keeping only the genomic regions and the corresponding reference haplotype matches that correspond to each individual ancestry. This step yielded three continental ancestry–specific sets of ChromoPainter2 local ancestry calls. The advantage of this approach is that it ensures that continental ancestry–specific haplotypes from admixed individuals are only compared with reference population haplotypes for the same ancestry, which does not always occur when ChromoPainter2 is run alone. Each set of continental ancestry–specific, local ancestry calls were then modeled using nonnegative least-squares (Mullen and van Stokkum 2012) to generate the final subcontinental ancestry fractions.

The f3 tree-based test was run in order to validate the African subcontinental ancestry inferences for admixed American populations, using the qp3pop program (Patterson *et al.* 2012). For each admixed American population with >10% average African ancestry, an f3 test statistical value was computed for the comparison f3(X; European, African), to distinguish the most likely African ancestral source population as Nigerian (Yoruba) or West African (Mende). The European outgroup populations used were British for the African American and African Caribbean admixed populations, and Spanish for the admixed populations of Chocó, Medellín, and Puerto Rico.

### Admixture timing analysis

The TRACTS program (Gravel 2012; Gravel *et al.* 2013) was used to infer the timing of admixture events in the admixed populations from the ancestry tracts defined by RFMix. For each admixed population, three possible orderings of admixture were evaluated with TRACTS: (1) European, Native American, and African; (2) European, African, and Native American; and (3) African, Native American, and European. For each ordering, TRACTS evaluated possible admixture timing from 14 to six generations ago, in 1000 bootstrap attempts. From the bootstrap attempts, the most likely series of admixture events was chosen to represent the population.

### Genetic *vs.* self-identified ancestry

Donors were asked to self-identify their ethnic origins according to six categories taken from the CIA World Factbook (CIA 2014). Each category corresponds to a single continental ancestry or a combination of two ancestries of African, European, and Native American: (1) African ($C_{A}$), (2) European ($C_{E}$), (3) Native American ($C_{N}$), (4) African-European ($C_{AE}$), (5) European-Native American ($C_{EN}$), and (6) African-Native American ($C_{AN}$). Individuals were free to choose one or more ethnic categories. For each individual, a self-identified ancestry fraction was calculated as a weighted average of the ancestry components among all of the ethnic groups chosen:

$${\text{Self}}_{\text{African}}=\frac{C_{A}+\frac{1}{2}C_{AE}+\frac{1}{2}C_{AN}}{\sum C_{i}},$$ (1)

$${\text{Self}}_{\text{European}}=\frac{C_{E}+\frac{1}{2}C_{AE}+\frac{1}{2}C_{EN}}{\sum C_{i}},$$ (2)

$${\text{Self}}_{\text{Native American}}=\frac{C_{N}+\frac{1}{2}C_{EN}+\frac{1}{2}C_{AN}}{\sum C_{i}},$$ (3)

i ∈{A, E, N, AE, EN, AN}.

### Data availability

Genome sequence variant data are available from the project resources listed in Table 1. Genotype data for Chocó are available by request, under the terms of a data use agreement managed by UTCH.

## Results

### Genetic ancestry in Chocó

**Figure 1:**
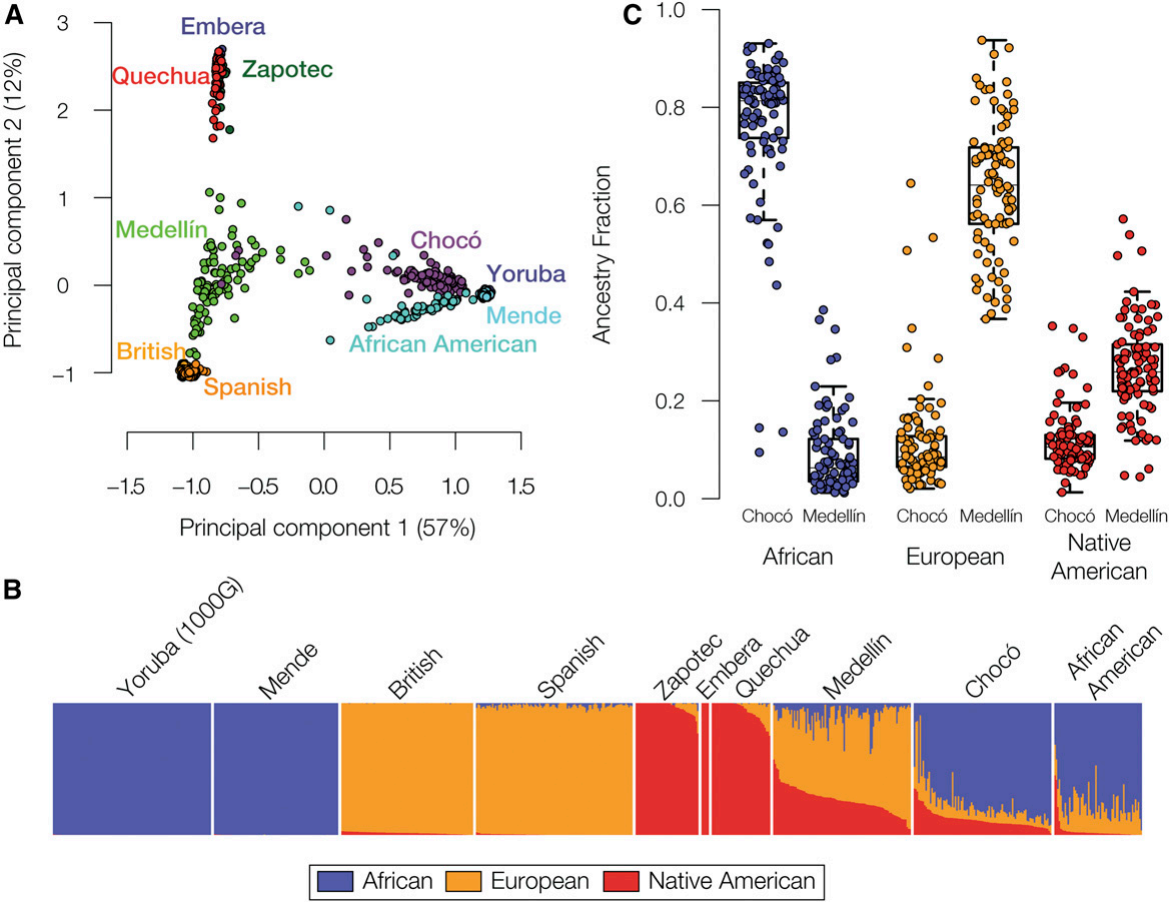
Comparison of continental ancestry for Chocó and Medellín. (A) Principal component analysis (PCA) projection of inter-individual genetic distance of admixed American populations, Chocó, Medellín, and African American, in relation to African, European, and Native American reference populations. (B) ADMXITURE plot showing genome-wide continental ancestry fractions for individuals from admixed American populations and global reference populations. Ancestry is shown as African (blue), European (orange), and Native American (red). The same ancestry color scheme is used throughout. (C) Box plots showing distributions of individuals’ continental ancestry fractions for the Chocó and Medellín populations.

Donor DNA samples from Chocó (Figure S2A in File S1) were genotyped and processed as described in the *Materials and Methods*. Donor genotypes were compared with local human reference populations from Colombia (Figure S2B in File S1) and to global human reference populations from Africa, Europe, and the Americas (Figure S2C in File S1), to characterize the genetic ancestry of Chocó. A total of 2050 individual genomes from 47 global populations were analyzed for this purpose (Table 1). Principal component analysis (PCA) of pairwise allele-sharing distances between individual genomes was used to visualize the relationship of the Chocó population to global reference populations (Figure 1A). As in our previous analysis (Medina-Rivas *et al.* 2016), individuals from Chocó group with African populations, consistent with the historically documented African ancestry of the region (Mosquera Córdoba 2014; Mosquera 2014), whereas Colombian individuals from the city of Medellín in the state Antioquia group most closely with the European populations. Nevertheless, results of the PCA indicate that Colombian populations from both Chocó and Medellín show three-way admixture, with varying contributions from African, European, and Native American ancestral source populations.

ADMIXTURE was used to infer continental ancestry fractions for the admixed Colombian populations from Chocó and Medellín via comparison with reference populations from Africa, Europe, and the Americas (Figure 1, B and C). The population of Chocó shows largely African in ancestry (76%), with the remainder being a nearly even split between European (13%) and Native American (11%) ancestry. The high levels of African genetic ancestry are consistent with the previously described historical and economic conditions that shaped the region as well as the modern concept of Chocó as home to a majority Afro-Colombian population. The population of Medellín has primarily European ancestry (75%), followed by Native American (18%) and African (7%) ancestry. The inferred continental admixture estimates for Chocó and Medellín are consistent with results from previous analyses (Homburger *et al.* 2015; Medina-Rivas *et al.* 2016; Rishishwar *et al.* 2015b). Individuals within both Chocó and Medellín vary widely with respect to their continental ancestry fractions. The African ancestry fraction shows the most inter-individual variation in Chocó (SD = 0.15), whereas individual European ancestry levels are most variable in Medellín (SD = 0.14).

As previously reported (Medina-Rivas *et al.* 2016), we also analyzed mitochondrial (mtDNA) and Y chromosome (Y-DNA) variants in order to evaluate continental ancestry along the maternal and paternal lineages for the population of Chocó (Table S1 in File S1). The majority of mtDNA (81%) and Y-DNA (74%) haplotypes have African origins, consistent with the results of the autosomal DNA analysis. The high percentages of both African mtDNA and Y-DNA haplotypes are consistent with historical records indicating that Chocó was populated with a fairly even ratio of female and male slaves: 46.6% female and 53.4% male from 1778 to 1808 (Appendix Table 13; Sharp 1976). The non-African mtDNA and Y-DNA haplotypes show evidence of sex-specific admixture contributions to the population of Chocó as has been previously reported for a number of Latin American populations (Bedoya *et al.* 2006; Bryc *et al.* 2010b; Carvajal-Carmona *et al.* 2000, 2003; Rishishwar *et al.* 2015b). All of the non-African mtDNA haplotypes (19%) have Native American origins, whereas all of the non-African Y-DNA haplotypes have either European (21%) or Middle Eastern/North African (5%) origins.

### Genetic *vs.* self-identified ancestry in Chocó

**Figure 2:**
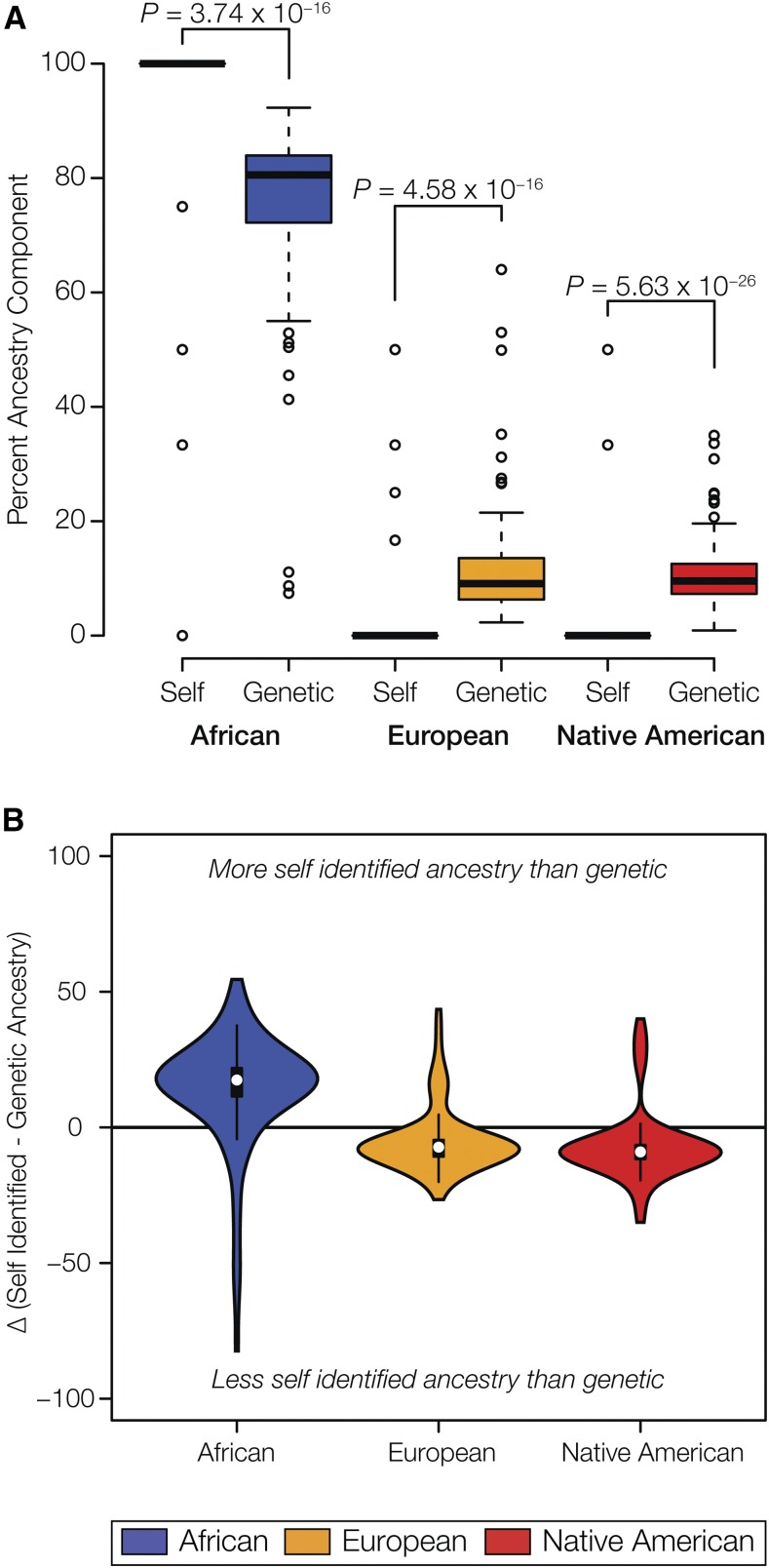
Genetic *vs.* self-identified ancestry in Chocó. (A) Box plots showing distributions of individuals’ self-identified *vs.* genetic continental ancestry fractions; *P*-values show the significance of the difference between the distributions. (B) Violin plots showing the distributions of the differences between self-identified and genetic continental ancestry.

Donors from Chocó were asked to self-identify their ethnicity, and their genetically defined ancestry fractions from the ADMIXTURE analysis were compared with their self-identified ancestries as described in the *Materials and Methods*. This analysis yielded distributions of genetic and self-identified ancestry percentages for each of the three continental groups – African, European and Native American – among the donors from Chocó (Figure 2A). Self-identified estimates of African ancestry are significantly higher than the genetically inferred African ancestry percentages, whereas the opposite trend is seen for both European and Native American ancestry components. In other words, individuals from the population of Chocó identify as more African, and less European/Native American, compared with what can be inferred from the ancestry analysis of their genome sequences (Figure 2B). The African self-identification of individuals from Chocó is consistent with the historical predominance of African slaves and free blacks in the region along with the conditions of economic and social isolation that have preserved its distinct Afro-Colombian character (Wade 1995).

### Local ancestry inference and admixture timing in Colombia

Given the distinct continental ancestry patterns seen for the populations of Chocó and Medellín, we wanted to compare the nature and timing of the admixture events that gave rise to the two populations. Local ancestry inference, whereby haplotypes are individually assigned continental ancestries, can be used to characterize the nature and timing of admixture. Specifically, the timing of genetic admixture can be inferred using the size distribution of ancestral haplotype tracts (Patterson *et al.* 2004). Ancestry tract sizes are expected to decay over time through recombination, such that on average shorter tracts indicate older admixture events whereas longer tracts indicate more recent admixture.

**Figure 3:**
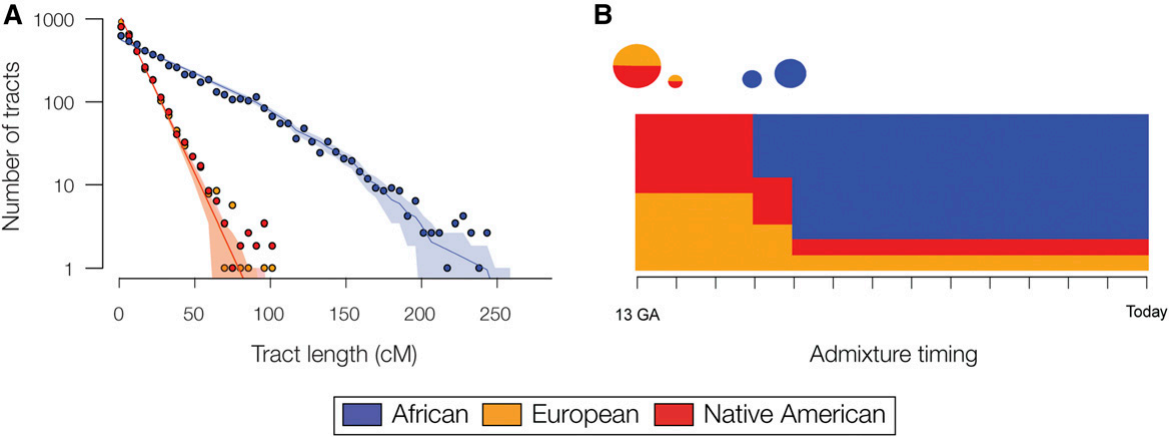
Modeling the timing of admixture events in Chocó. (A) Observed (points) and predicted (solid line) ancestry tract size distributions; the shaded areas represent 95% confidence intervals. (B) Admixture event timings are shown together with ancestry proportions. Each inferred admixture event is indicated by a circle, which is scaled according to the size of the contribution to the population and also shows the relative ancestry proportions. The *y*-axes of the charts show the inferred continental ancestry fractions, and the *x*-axes show time as the number of generations ago (GA).

African, European and Native American haplotype tracts were characterized for individuals from Chocó and Medellín using the program RFMix (Maples *et al.* 2013). As a control, the global ancestry fractions produced by the RFMix analysis were compared with the results generated by ADMXITURE for the same individuals (Figure 1B), yielding very high correlations (Figure S3 in File S1). The ancestry tracts for Chocó and Medellín were then analyzed with the program TRACTS (Gravel 2012; Gravel *et al.* 2013), which models the size of the ancestry tracts in a population and fits the most likely sequence of admixture timing events to the observed tract size distributions. The most likely model generated for Chocó suggests an early, single admixture event between European, likely Spanish, and Native American populations, followed by a later, larger African admixture event (Figure 3). Medellín shows a similar order of admixture events, with initial European-Native American admixture followed by subsequent African admixture, albeit more closely spaced in time (see *Discussion* on pg. 5 of the Supplement and Figure S4 in File S1).

### Subcontinental ancestry origins in Colombia

The results described thus far deal with the clearly distinct continental ancestry and admixture patterns that can be observed for Chocó and Medellín. However, genetic analysis also allows for the characterization of subcontinental ancestry origins for individuals from admixed populations. In other words, it is possible to more precisely define the specific populations within Africa, Europe and the Americas that mixed to form modern Colombian populations. We also wanted to quantify the relative contributions of different subcontinental ancestries to the other admixed American populations analyzed here. To do so, we developed and applied a novel approach to subcontinental ancestry inference that combines haplotype continental ancestry tracts generated by the RFMix program with fine-scale ancestry inference utility of the ChromoPainter2 algorithm (see *Materials and Methods*). The advantage of our novel, hybrid approach to subcontinental ancestry inference is that it allows the ChromoPainter2 program to tease apart the subcontinental ancestry contributions for each distinct continental ancestry fraction separately.

**Figure 4:**
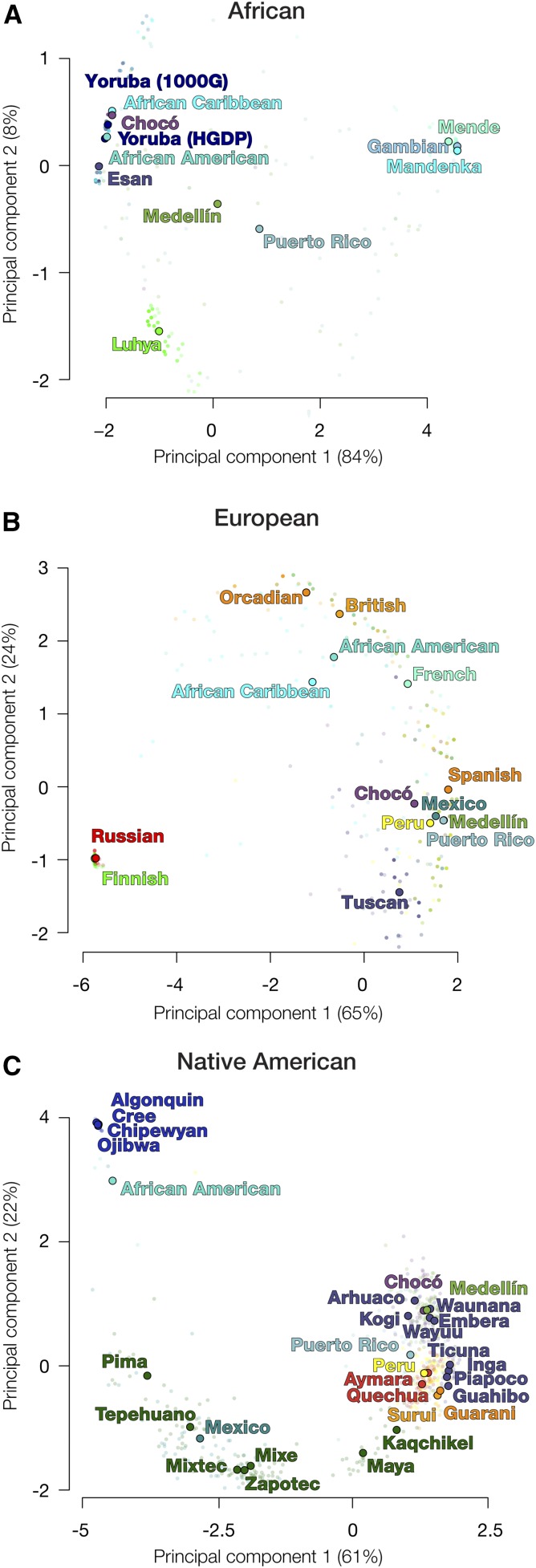
Ancestry-specific principal component analysis (PCA) for admixed American and global reference populations. PCAs are shown separately for (A) African, (B) European, and (C) Native American ancestry fractions. The locations of the dots correspond to population-specific centroids estimated from all individuals within each population. Population descriptions can be found in Table 1.

For each continental ancestry, we used ChromoPainter2 to paint admixed individuals using the ancestral source populations shown in Table 1 as references and to paint each ancestral individual using all the other ancestral individuals. For each admixed individual, ChromoPainter2 yields a painting vector that describes how often that individual is painted as each distinct reference population. The ChromoPainter2 output was processed to mask any extra-continental haplotypes identified by RFMix, yielding continental-specific paintings of both admixed and ancestral individuals. From these painting vectors, we found the Spearman rank-correlations between all pairs of individuals within each continental ancestry, and visualized these using PCA (Figure 4).

PCA of African haplotype paintings revealed that the African ancestry of Chocó is most closely related to Nigerian populations and distinct from the African ancestry of Medellín (Figure 4A). The African ancestry of Medellín is most similar to that of the Puerto Rico population and intermediate between Nigerian and other West African populations. The European fractions of the admixed Latin American populations were all largely similar to each other and closest to the Spanish population (Figure 4B), whereas the European fractions of the African American and African Caribbean populations were more closely related to the British and French populations. The Native American PCA results show the Chocó and Medellín populations grouping together with the Colombian Native American populations: Embera, Waunana, Arhuaco, Kogi and Wayuu (Figure 4C), and being most closely related to the Embera and Waunana. The Native American fractions of the African American, Mexican and Peruvian populations also group according to their geography, with North American, Mesoamerican and Andean Amerindian populations, respectively. The Native American ancestry of Puerto Rico is relatively distant from the other South American populations, though closest to the Andean groups, similar to what has been previously shown (Moreno-Estrada *et al.* 2013). Using the RFMix-masked genotypes, we also constructed phylogenetic trees for each of the three continental ancestries using the population-specific allele frequencies in the unmasked regions. These trees are largely congruent with the ancestry-specific PCA results, as well as a previous analysis of Native American genetic ancestry (Reich *et al.* 2012), and place the Chocó and Medellín Native American ancestry components as a sister clade to the Embera and Waunana groups (Figure S5 in File S1).

**Figure 5:**
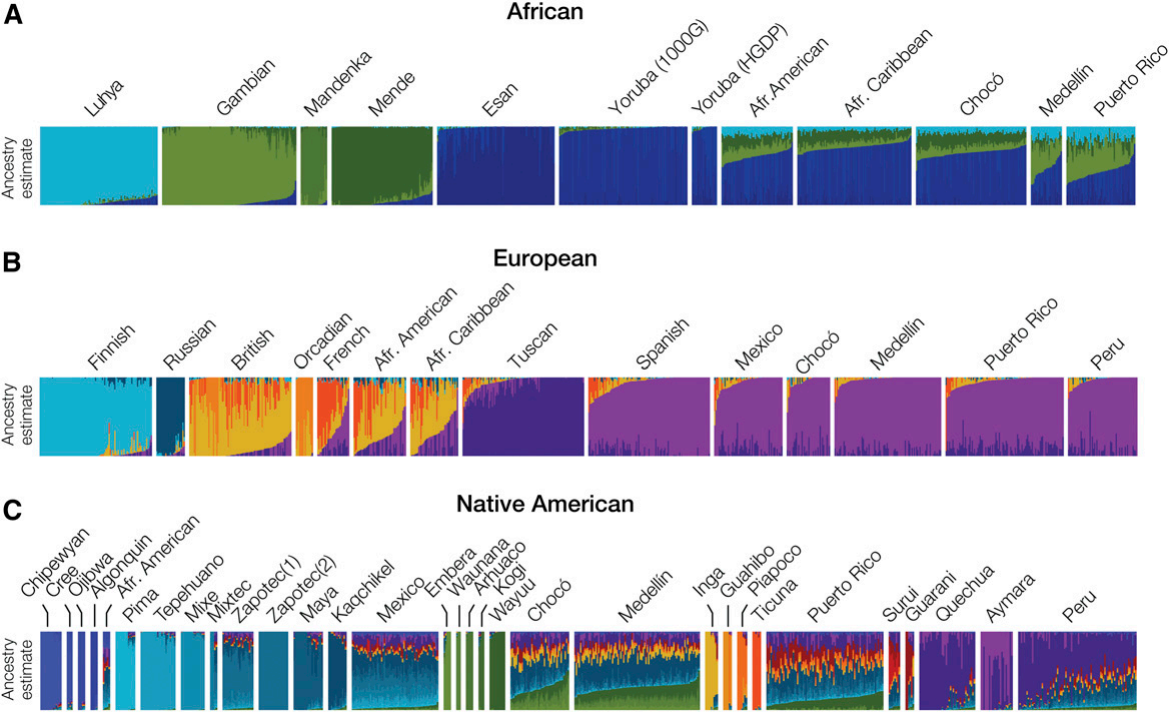
Subcontinental ancestry of admixed American and global reference populations. Subcontinental ancestries for admixed American populations were inferred separately using (A) African, (B) European, and (C) Native American reference populations. ChromoPainter2 was used to paint reference and admixed individuals as mosaics of reference individuals. The estimated subcontinental ancestry derived from the reference populations is shown for each individual. Population descriptions can be found in Table 1.

To further quantify the subcontinental ancestries of admixed American populations, we obtained a separate ChromoPainter2 painting for each of the three continental ancestries: African, European, and Native American. Across the whole genome of any individual, the sum of matches from each ancestral population yields a vector of painting frequencies as shown in the top row of each ancestry-specific panel in Figure S6 in File S1. The observed painting frequencies can, in turn, be used to model both admixed and ancestral individuals as a linear combination of ancestral populations in order to quantify the subcontinental ancestry contributions. To do this, we used non-negative least squares (NNLS) to find the best fit of ancestral populations in every individual as shown in Figure 5. The resulting values can be averaged across individuals to quantify population-specific subcontinental ancestry.

For the admixed American populations with significant African ancestry, African American (62%), African Caribbean (74%), and Chocó (66%) all show relatively greater shared ancestry with Nigerian populations compared with Medellín (46%) and Puerto Rico (43%) (Figure 5A). Conversely, Medellín and Puerto Rico show greater subcontinental ancestry contributions from other nearby West African populations, Sierra Leone and Gambia, compared with Chocó. All five of the Latin American populations analyzed here have nearly uniformly Spanish subcontinental European ancestry: Chocó (85%), Medellín (92%), Mexico (83%), Peru (88%) and Puerto Rico (83%) (Figure 5B), whereas the African American and African Caribbean populations show a mix of British and French ancestries. For Native American subcontinental ancestry, Chocó and Medellín produced very similar paintings, indicating that the ancestral Native American populations that contributed to these two diverse, modern Colombian populations were very closely related (Figure 5C). There are five Native American populations in particular that appear as the most likely ancestral source populations for both Chocó and Medellín: Embera, Waunana, Arhuaco, Kogi and Wayuu. All five of these Native American populations are found in close proximity within Northern and Western Colombia (Figure S2B in File S1). We obtained qualitatively identical results for all of these subcontinental ancestry assignments via analysis with ADMIXTURE (Figure S7, Figure S8, and Figure S9 in File S1).

**Figure 6:**
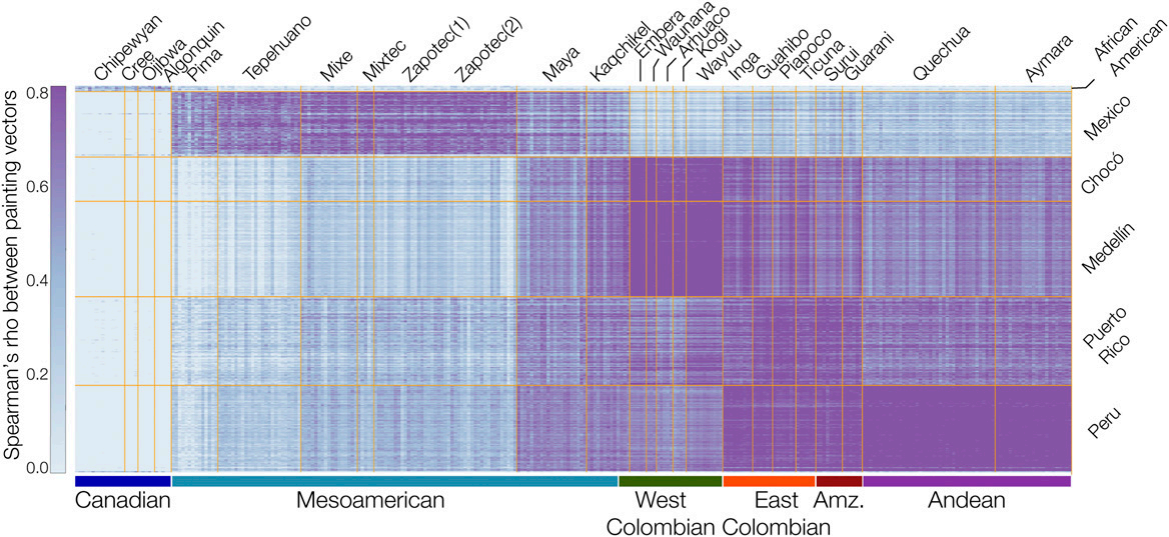
Native American subcontinental ancestry of admixed American populations. Spearman rank correlations (color coded as seen in the key) are shown for ChromoPainter2 painting vectors between all pairs of individuals from Native American reference populations (*x*-axis) and admixed American populations (*y*-axis). Native American reference populations are grouped according to their geographic origins as shown below the plot (Amz. – Amazonian). Population descriptions can be found in Table 1.

The Native American reference populations analyzed here show high levels of population structure with far greater within *vs.* between group similarity levels. This is true for even closely related pairs of reference populations, such as the Arhuaco and Kogi or the Embera and the Waunana, which show far higher levels of within *vs.* between group similarity compared with other closely related reference population pairs, *e.g.*, the British and Orcadian populations. This raises the possibility that admixed populations with ancestries not derived specifically from one of the reference Native American populations may incorrectly identified using the NNLS modeling. To control for this possibility, we used an alternative approach to identify the most likely Native American source populations for Chocó and Medellín. To do this, we found the Spearman rank-correlation of painting vectors between admixed individuals and reference individuals for each continental ancestry (Figure 6). This analysis yields six groups of Native American ancestral source populations that are differentially distributed among the admixed American populations. As was seen with the PCA analysis (Figure 4C), the geographic origins of the ancestral populations correspond extremely well with the locations of the modern admixed populations. Chocó and Medellín show very similar patterns with the highest levels of similarity seen for Embera, Waunana, Arhuaco, and Kogi. The Mexico and Peru populations show the highest levels of similarity with Mesoamerican and Andean Native American populations, respectively. The African American population is the only one that shows similarity to the Native American reference populations from Canada. The Puerto Rico population shows the lowest overall similarity to the Native American reference populations analyzed here, with highest similarity to the East Colombian Native American and the Amazonian populations. As was the case for the hybrid RFMix-ChromoPainter2 approach, we obtained qualitatively identical results for all of these subcontinental ancestry assignments taking a similar approach with the ADMIXTURE (Figure S7, Figure S8, and Figure S9 in File S1).

We carried out the same correlation analysis for African and European subcontinental ancestry. Consistent with the NNLS results, the African ancestries of the African American, African Caribbean, and Chocó populations generally correlate best with Nigerian individuals, whereas the Medellín and Puerto Rico populations were more heterogeneous, correlating with Nigerian as well as other West African populations (Figure S10 in File S1). The European ancestry of admixed African American and African Caribbean populations correlates best with the British, Orcadian, and French populations, whereas Latin American populations correlate best with Spanish individuals (Figure S11 in File S1).

## Discussion

### Two waves of forced African migration to Colombia

**Figure 7:**
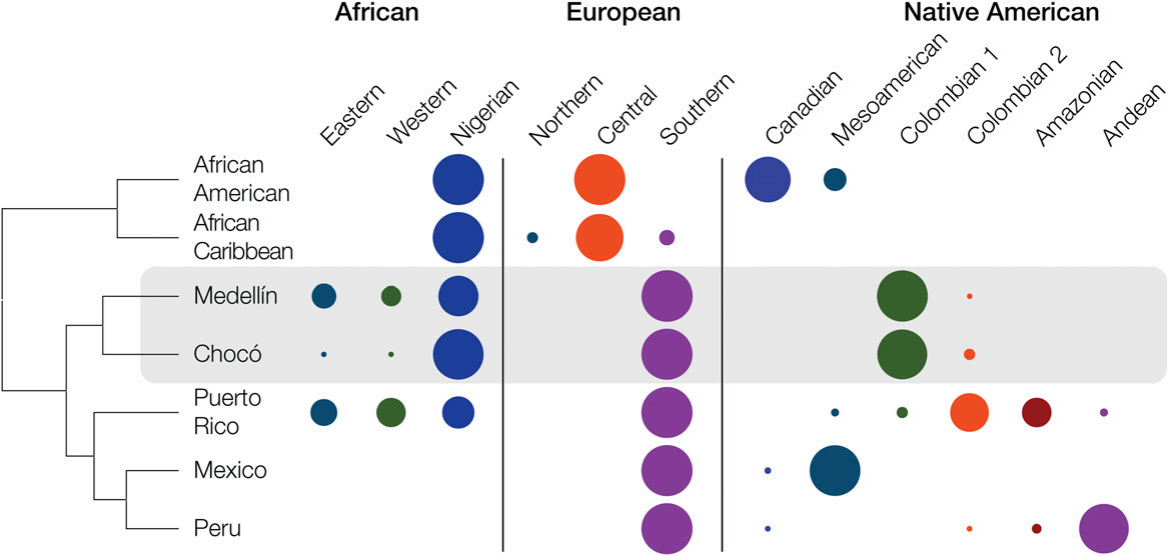
Subcontinental ancestry profiles of admixed American populations. African, European and Native American ancestries are broken down into their major subcontinental fractions as shown. The sizes of the subcontinental ancestry circles represent the relative contributions to each admixed American population. The dendogram shows the relationships among the admixed American populations based on their subcontinental ancestry profiles. Population descriptions can be found in Table 1.

The application of our novel method for subcontinental ancestry analysis allowed for the characterization of fine scale differences in the genetic ancestry patterns of Colombian and other admixed American populations. The populations of Chocó and Medellín show very similar patterns of European and Native American subcontinental ancestry, grouping them together to the exclusion of the other admixed populations analyzed here (Figure 4, B and C and Figure 5, B and C). However, there are subtle differences in the patterns of African subcontinental ancestry that distinguish the population of Chocó from that of Medellín (Figure 4A and Figure 5A). Chocó has an almost exclusively Yoruba African ancestry, whereas Medellín shows high levels of Yoruba ancestry along with relatively more ancestry from other West African populations in Gambia and Sierra Leone (Figure 4A, Figure 5A, and Figure 7). In light of these unexpected results, we also used the f3 tree-based statistic in an effort to further validate the most likely African source populations for Chocó and Medellín. The f3 test also shows the Yoruba population as the best match for Chocó, whereas Medellín shows very similar f3 test statistic values for the Yoruba population and the Mende population from Sierra Leone in West Africa (Table S2 in File S1). Thus, the results of the f3 analysis are entirely consistent with both the ancestry-specific PCA and the RFMix-ChromoPainter2 results showing the differences in African subcontinental ancestry for the two Colombian populations. All of the inferred African ancestral source populations for Chocó and Medellín correspond to groups that spread throughout sub-Saharan Africa via the Bantu expansion ∼4000 yr ago (Bryc *et al.* 2010a), consistent with historical records of the transatlantic slave trade.

Interestingly, the differences in subcontinental African ancestry observed for Chocó *vs.* Medellín correspond to two distinct eras of forced African migration to Colombia (Rishishwar *et al.* 2015a; Rodriguez 2008; Maya Restrepo 2005). Historical records of *trans*-Atlantic slave voyages point to a first wave of migration from West Africa to Colombia in the mid-16th century. The geographic regions and populations enumerated in this particular set of records correspond best to the subcontinental African ancestry patterns seen for Medellín. A second, later wave of African migration to Colombia occurred from the mid-17th to the mid-19th centuries. These voyages originated in Nigeria and surrounding areas, and included primarily individuals of Yoruba ancestry. Accordingly, the subcontinental African ancestry pattern seen for Chocó is more consistent with this second wave of African migration.

Afro-descendant populations in Colombia are found primarily along the Atlantic (Caribbean) and Pacific coast lines (Hernández Romero 2005). The African subcontinental ancestry pattern of Medellín groups it more closely with Puerto Rico, from the Caribbean, than with Chocó (Figure 4A and Figure 5A). This suggests the possibility that Afro-Colombian populations from the Caribbean coastal area of the country have a distinct ancestry profile compared with those from the Pacific coast, and the more ancient African ancestry component seen for Medellín may correspond better with Caribbean Afro-Colombian populations. Further comparative studies of distinct Afro-Colombian populations from these two regions could be used to investigate this possibility.

### Genetics of mestizaje *vs.* multiculturalism in Colombia

At first glance, when the overall genetic ancestry of Afro-descendant (Chocó) and Mestizo (Medellín) populations from Colombia are compared, they appear to be quite different. Unsurprisingly, given their respective continental ancestry profiles, Chocó groups closely with African populations whereas Medellín groups more closely with European populations and other admixed Latin American populations (Figure 1). However, when all three continental ancestry components are considered separately, we observe substantial shared subcontinental ancestry between the populations of Chocó and Medellín. In particular, when direct comparisons between members of these two populations are performed on haplotypes that come from within the same ancestry groups – African, European or Native American – Chocó and Medellín group together to the exclusion of all other admixed American populations (Figure 4 and Figure 7). On the one hand, this could be expected given the pre-Columbian (for the Native American component) and colonial (for the African and European components) history of the Americas. On the other hand, the shared ancestry of these two Colombian populations can also be considered to have bearing on issues related national identity and consciousness.

Specifically, we consider that the shared subcontinental ancestry between Colombian population groups can be taken to have implications for the roles of ethnic and cultural identity in the country (Wade *et al.* 2014). *Mestizaje* refers to the intentional mixing of different population groups, to create “*la nación mestiza*” or the mixed nation (Chavez and Zambrano 2006). The theory and practice of *mestizaje* were critical dimensions of nation building in Latin America; *mestizaje* celebrated unity of purpose and identity via ethnic and cultural blending. In some sense, the philosophy of *mestizaje* can be considered as diametrically opposed to historical prohibitions against racial mixing in North America. Nevertheless, *mestizaje* has been criticized as an ideology of exclusion that reinforces racial hierarchies and encourages homogenization, leading to a primarily Mestizo national identity that leaves little or no room for indigenous or Afro-Latino identities (Wade 1995). Multiculturalism stands in opposition to *mestizaje* with respect to its emphasis on an increased recognition of ethnic and cultural diversity, or pluralism, in Latin American societies (Chavez and Zambrano 2006). The move toward an explicitly multicultural identity, which to some extent is derived from a more Anglo-American worldview, is a relatively recent trend in Latin America and is considered by some as a more inclusive ideology that is better able to accommodate societal diversity. On the other hand, critics of multiculturalism point to its potential to manifest as a divisive ideology when taken to logical extremes, such as its expression in identity politics (Barry 2002).

Results obtained in this study indicate that Afro-Colombian and Mestizo Colombian populations, which appear to be extremely different at first glance, were in fact formed from similar ancestral source populations, albeit via different relative frequencies of African, European and Native American ancestors. This is particularly true for the Native American ancestry components of the two populations, which are virtually identical, as may be expected given what is known about the distribution of Native American populations in the region prior to Spanish colonization. These results on genetic ancestry have implications for conceptualizing Colombia as a true “*nación mestiza*” given the underlying unity of genetic ancestry between seemingly diverse populations, which would otherwise occupy exclusive identities according to an explicitly multicultural (pluralistic) worldview.

### Ethnic self-identity and genetic ancestry in Chocó *vs.* Medellín

We previously used comparisons of genetic ancestry with ethnic self-identity captured from census data to show that the population of Medellín identifies as having far more European ancestry than can be seen from analysis of their genome sequences, which show substantial admixture with Native American and African ancestral populations (Rishishwar *et al.* 2015b). We speculated that the distinction between the almost exclusively European self-identity of individuals from Medellín, and their observed levels of genetic admixture, could be related to the concept of *blanqueamiento* (literally whitening), an ideology of social improvement via a progressive whitening of the population whereby whiteness is held as a social ideal that should be aspired to (Telles and Flores 2013). For this study, we tried to derive a more direct measure of ethnic self-identity in Chocó by having sample donors choose from among census-based ethnic categories, each of which corresponds to either a single continental ancestry or a combination of two ancestries. Self-identified ancestry fractions were then computed from donor selections and compared with their genetic ancestry estimates (Figure 2). Interestingly, Chocó shows the opposite pattern of Medellín; on average, individuals from Chocó identify as having more African ancestry, and less European or Native American ancestry, than can be gleaned from analysis of their genome sequences. These results point to a strong affinity with African heritage in Chocó and are consistent with the rich cultural traditions of the region (Carrillo 2012; Jimeno *et al.* 1995; Mosquera Córdoba 2014; Mosquera 2014; Wade 1995).

### Current limitations and future directions

Any study of the genetic ancestry of admixed American populations, such as we report here, will necessarily be limited by the reference panel of putative ancestral source populations that are used. For the current study, we were able to identify the most closely related populations from the global reference panels that we used with certainty. However, we cannot formally rule out the possibility that there are other populations, not included in our current reference panel, which may be more closely related to the admixed populations studied here. Considering the populations of Chocó and Medellín, this is not likely to be much of a problem for European or Native American ancestry, since our reference panels cover the most likely ancestral source populations for these continental groups quite well. However, there may be African populations not included in our reference genome panel that show greater similarity to the admixed Colombian populations, than is seen for the African populations that we currently employ as putative ancestral source populations. This is particularly true for African populations from the southwest part of the continent, corresponding to modern populations of Congo and Angola, from which historical records indicate that many Africans were forcibly taken to Colombia (Figure S1 in File S1). Future studies of admixed Latin American populations, particularly Afro-Latino populations, would benefit from a more robust collection of African reference genomes.

A corollary caveat for our analysis regards the interpretation of the results of the subcontinental ancestry analysis. For example, our finding of predominantly Nigerian (Yoruban) subcontinental ancestry for the African ancestry component in the population of Chocó is best understood as shared ancestry between present-day Nigerians and the African ancestors of the modern population of Chocó.

### Conclusions

Our initial studies of the population of Chocó have revealed a number of insights regarding Afro-Colombian genetic ancestry, and in so doing have expanded the notion of what it means to be Colombian from a genetic point of view. Nevertheless, there are a number of diverse Afro-Colombian populations found throughout the country, and further comparative studies among these populations should tell us even more about the genetic ancestry of Colombia. For instance, an Afro-Colombian population from the community Palenque de San Basilio near the Caribbean coast shows a distinct pattern of African ancestry compared with Chocó or Medellín with individuals most closely related to Yombe speakers from the Republic of the Congo (Ansari-Pour *et al.* 2016).

A major point that we would like to emphasize from this study is the extent of shared subcontinental ancestry seen for the populations of Chocó and Medellín, which at first glance appeared to be very distantly related. As we have discussed previously, this shared genetic legacy underscores the biological reality of a common, unifying identity that binds the country, consistent with the aforementioned notion of “*la nación mestiza*” (Chavez and Zambrano 2006).

## Supplementary Material

Supplemental material is available online at www.g3journal.org/lookup/suppl/doi:10.1534/g3.117.1118/-/DC1.
