# Supplementary material for: A Comparative Analysis of Genetic Ancestry and Admixture in the Colombian Populations of Chocó and Medellín
Source: G3 (Bethesda). 2017 Aug 28;7(10):3435–47. doi: 10.1534/g3.117.1118 (PMC5633392; doi:10.1534/g3.117.1118)
Supplement: Supplementary file 1 [file 3435FileS1.pdf]

Supplementary Material for:

A comparative analysis of genetic ancestry and admixture in the Colombian populations of Chocó and Medellín

Andrew B. Conley[1,2], Lavanya Rishishwar[1,2], Emily T. Norris[1,2,3], Augusto Valderrama-Aguirre[2,4], Leonardo Mariño-Ramírez[2,5], Miguel A. Medina-Rivas[2,6] and I. King Jordan[1,2,3,*]

[1]Applied Bioinformatics Laboratory, Atlanta, GA, USA, [2]PanAmerican Bioinformatics Institute, Cali, Valle del Cauca, Colombia, [3]School of Biological Sciences, Georgia Institute of Technology, Atlanta, USA, [4]Biomedical Research Institute, Universidad Libre, Cali, Valle del Cauca, Colombia, [5]National Center for Biotechnology Information, National Library of Medicine, National Institutes of Health, Bethesda, MD, USA, [6]Centro de Investigación en Biodiversidad y Hábitat, Universidad Tecnológica del Chocó, Quibdó, Chocó, Colombia

CONTENTS

Supplementary Figures S1-S13

Supplementary Tables 1 & 2

Admixture timing in Medellín (page 5)

Geographical population structure in Chocó (page 13-16)

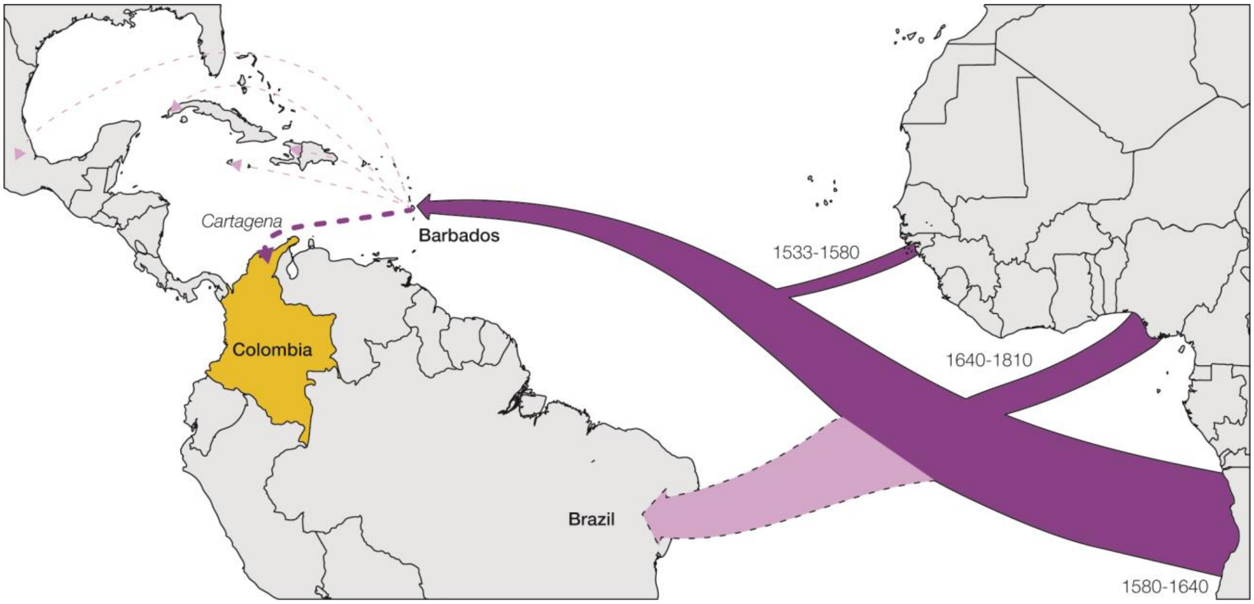

Supplementary Figure S1 The transatlantic slave trade and Colombia. Cartagena was the main port of entry for African slaves in Colombia. There were three eras of documented forced migration from Africa to Colombia: (1) 1533-1580, West Africa (Gambia, Guinea, Senegal, Sierra Leone), 6,000 individuals, (2) 1580-1640, Southwest Africa (Angola, Congo), 340,000 individuals, and (3) 1640-1810 West-Central Africa (Ghana, Nigeria, Cameroon), 200,000 individuals.

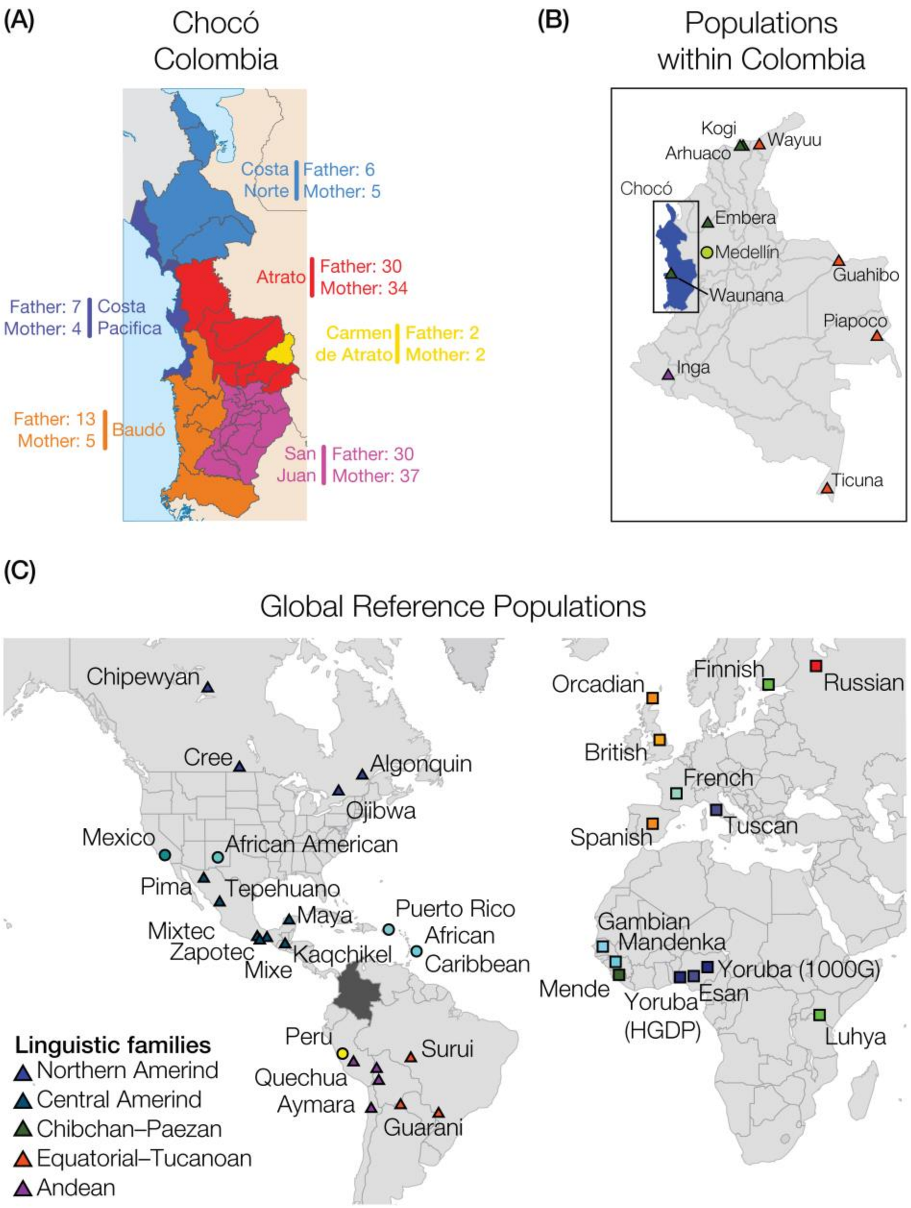

Supplementary Figure S2 Geographic origins of the samples analyzed here. (A) Parental origins of sample donors from Chocó for six geographic regions. (B) Admixed Colombian populations from Chocó and Medellín (circles) along with Native American reference populations from Colombia (triangles). (C) Global reference populations and admixed American populations. Old World populations from Europe and Africa are shown as squares, New World Native American populations are shown as triangles, and admixed American populations are shown as circles. Native American reference populations are color coded according to their linguistic families[21].

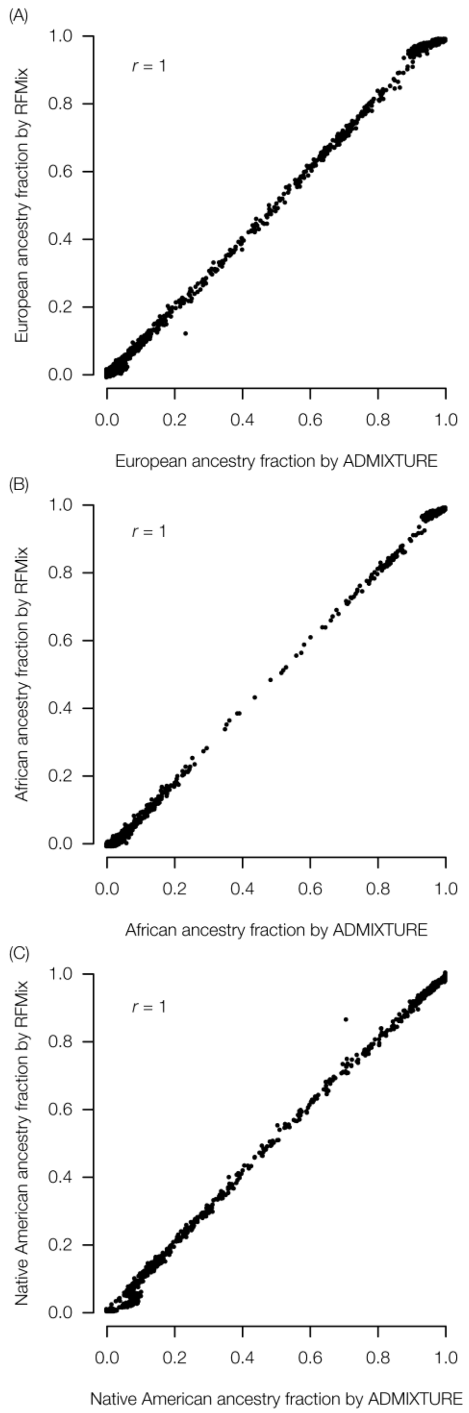

Supplementary Figure S3 Comparison of ancestry fractions estimated by ADMIXTURE (global) versus RFMix (local). Correlation between ADMIXTURE and RFMix ancestry estimates for individuals analyzed here are shown for (A) European, (B) African and (C) Native American ancestry. Pearson correlation r-values are shown for each ancestry. Genome-wide continental ancestry fractions were inferred for all individuals using the program ADMIXTURE, which estimates global ancestry fractions, and by summing the lengths of local ancestry-specific tracts (haplotypes) inferred from RFMix.

ADMIXTURE TIMING IN MEDELLÍN

The nature of the admixture events in Medellín is also distinguished by the relatively higher amounts of European and Native American ancestry compared to Chocó, consistent with the results of our global ancestry analysis. The results of the TRACTS analysis for Medellín are also consistent with a previous analysis of 1KGP data for this population (Moreno-Estrada et al. 2013). In particular, the distribution of ancestry tracts that we observe for Medellín are very similar to what was shown by Moreno-Estrada et al., including a broad tail of a few longer ancestry tracks, particularly for Native American ancestry, which do not the fit the admixture timing model as well as the African and European tracts. The previous study also used a slightly more complex model that entailed multiple pulses of admixture; although, the additional parameters introduced for the more complex model only yield a slightly better fit. In any case, both our results and the previous analysis indicate that the populations of Chocó and Medellín were formed by high levels of early admixture between continental source populations followed by a longer period where the relative continental ancestry fractions remained constant.

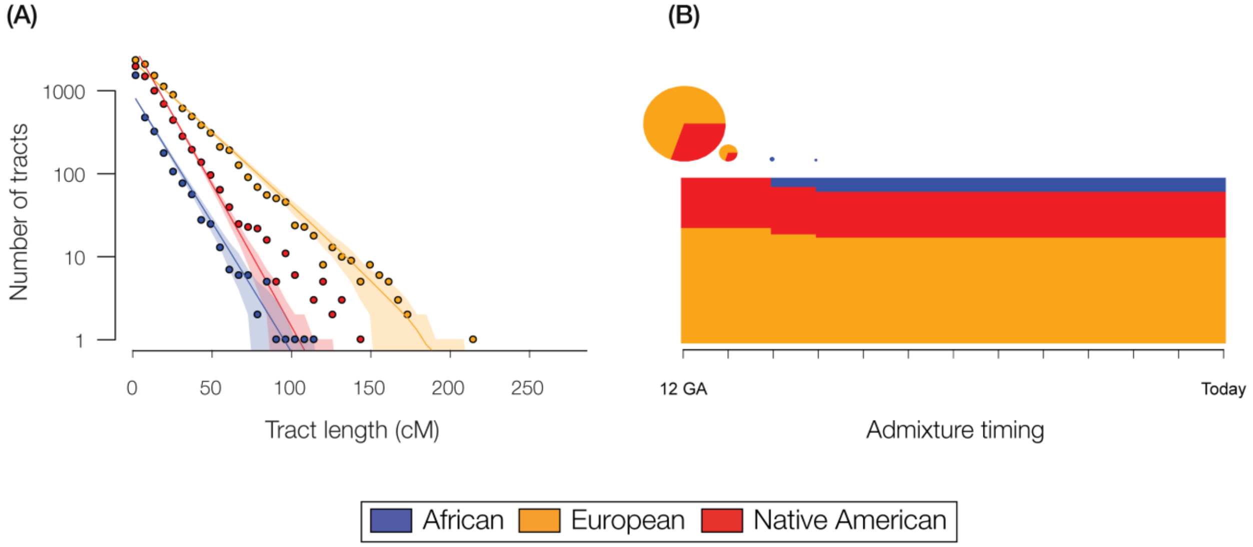

Supplementary Figure S4 Modeling the timing of admixture events in Medellín (lower panel). (A) Observed (points) and predicted (solid line) ancestry tract size distributions. The shaded areas represent 95% confidence intervals. (B) Admixture event timings are shown together with ancestry proportions. Each inferred admixture event is indicated by a circle, which is scaled according to the size of the contribution to the population and also shows the relative ancestry proportions. The y-axes of the charts show the inferred continental ancestry fractions, and the x-axes show time as the number of generations ago (GA).

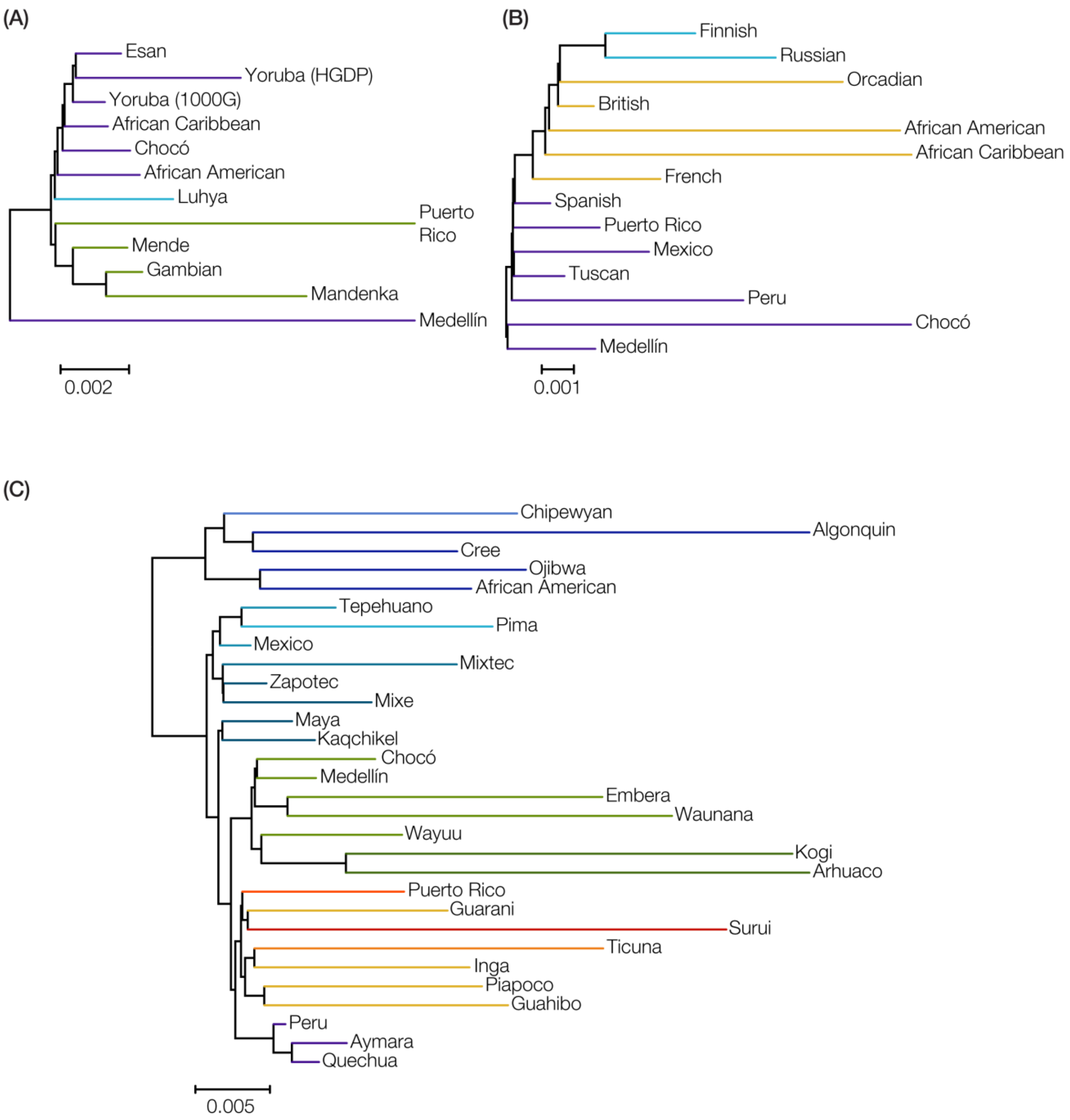

Supplementary Figure S5 Phylogenetic analysis of continental ancestry groups. RFMix was used to identify ancestry tracts for each of the three continental ancestry groups – (A) African, (B) European and (C) Native American – and to mask extra-continental tracts among all individuals analyzed here. Then, for the tracts corresponding to each individual continental ancestry group, average pairwise distances between all pairs of populations were computed using Nei's standard genetic distance. Neighbor-joining phylogenies were reconstructed for each continental ancestry-specific pairwise distance matrix.

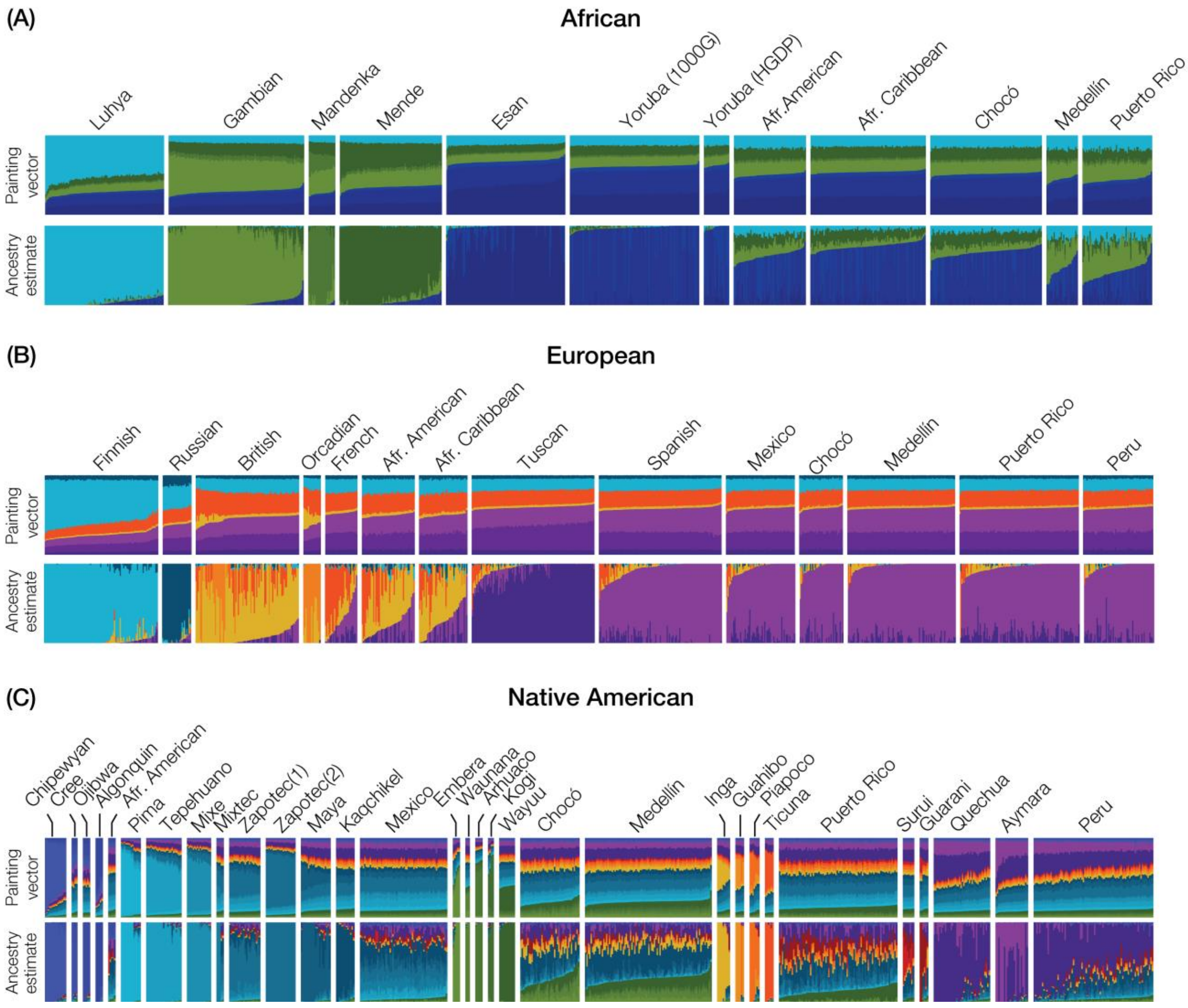

Supplementary Figure S6 Subcontinental ancestry of admixed American and global reference populations. Subcontinental ancestries for admixed American populations were inferred separately using (A) African, (B) European and (C) Native American reference populations. The ChromoPainter2 painting vectors for reference and admixed individuals are shown for each continental ancestry (top panels). The estimated sub-continental ancestry derived from the reference populations is shown for each individual (bottom panel).

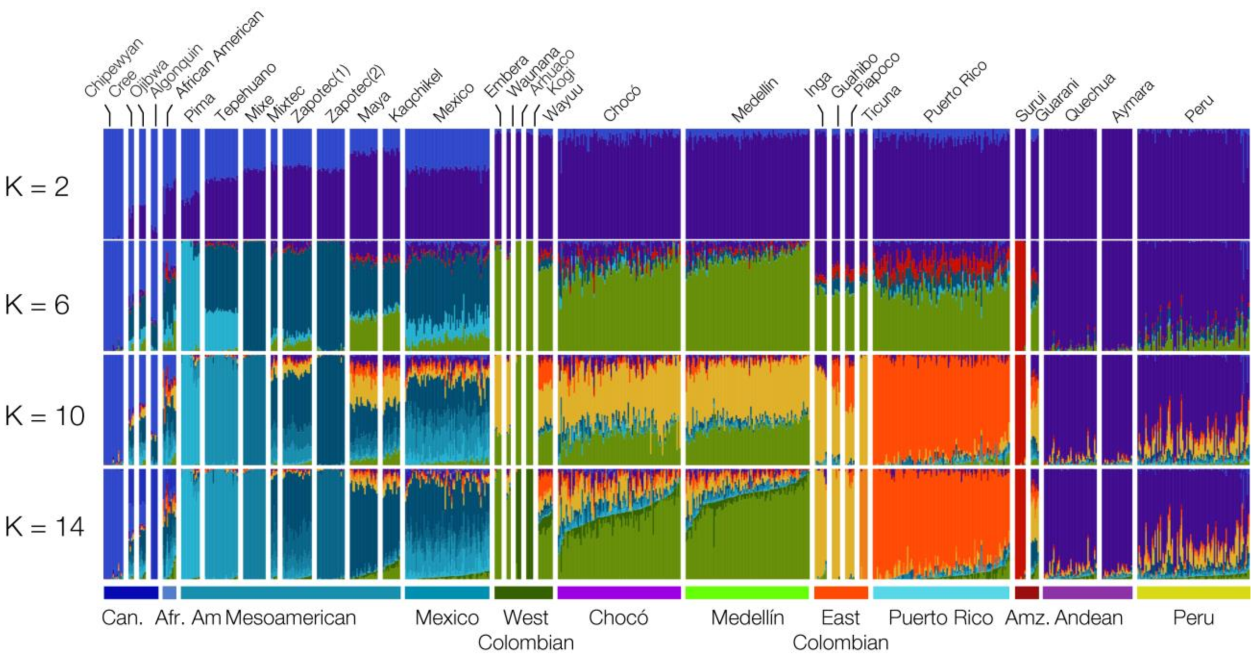

Supplementary Figure S7 ADMIXTURE analysis of Native American ancestry. African and European ancestry-specific tracts (haplotypes) of reference and admixed individuals were masked using RFMix, and the program ADMIXTURE was then used to characterize the remaining Native American regions. The values of K correspond to the number of clusters used in the ADMIXTURE analysis.

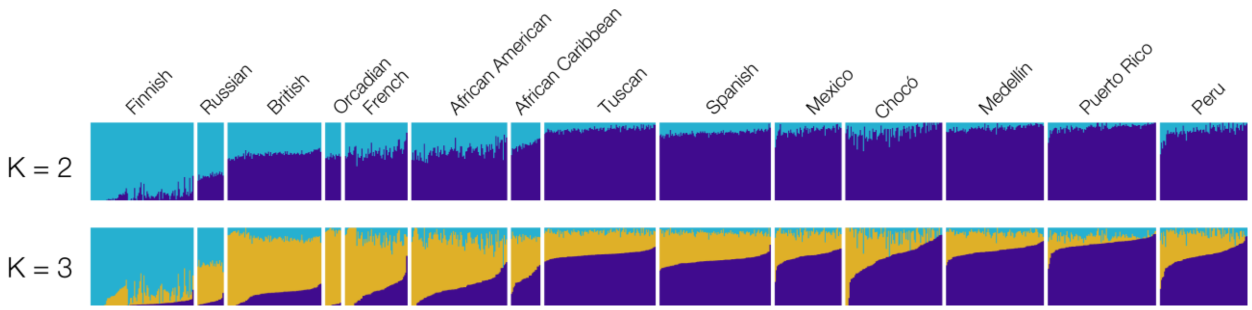

Supplementary Figure S8 ADMIXTURE analysis of European ancestry. African and Native American ancestry-specific tracts (haplotypes) of reference and admixed individuals were masked using RFMix, and the program ADMIXTURE was then used to characterize the remaining European regions. The values of K correspond to the number of clusters used in the ADMIXTURE analysis.

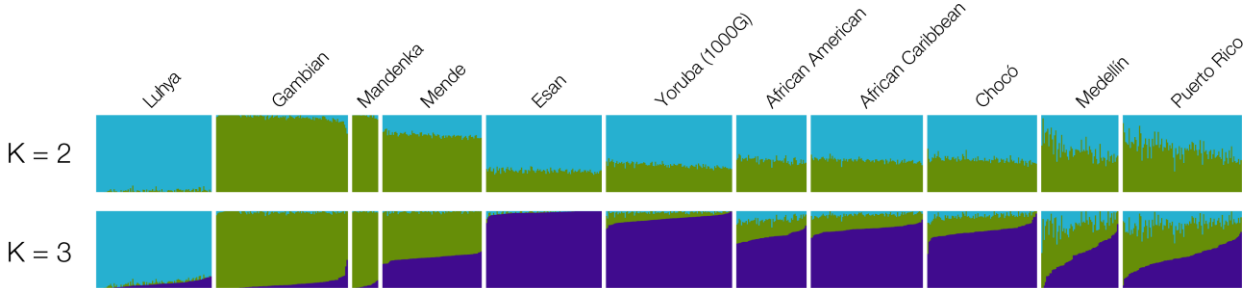

Supplementary Figure S9 ADMIXTURE analysis of African ancestry. European and Native American ancestry-specific tracts (haplotypes) of reference and admixed individuals were masked using RFMix, and the program ADMIXTURE was then used to characterize the remaining African regions. The values of K correspond to the number of clusters used in the ADMIXTURE analysis.

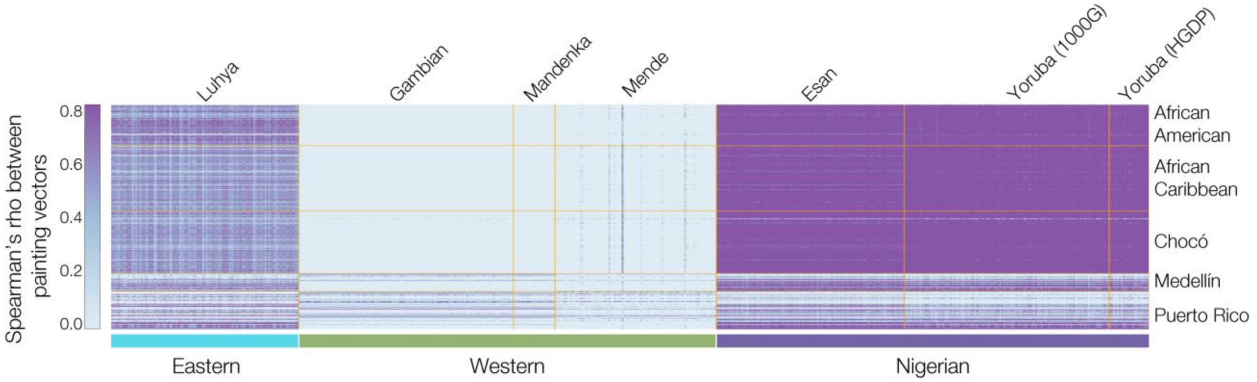

Supplementary Figure S10 African subcontinental ancestry of admixed American populations. Spearman rank correlations for ChromoPainter2 painting vectors (color-coded as seen in the key) are shown for all pairs of individuals from African reference populations (x-axis) and admixed American populations (y-axis). African reference populations are grouped according to their geographic origins as shown below the plot.

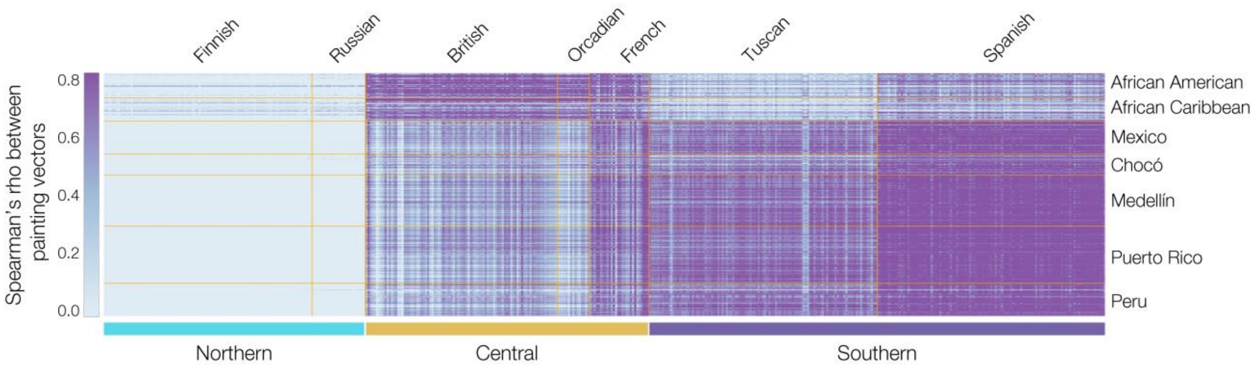

Supplementary Figure S11 European subcontinental ancestry of admixed American populations. Spearman rank correlations for ChromoPainter2 painting vectors (color-coded as seen in the key) are shown for all pairs of individuals from European reference populations (x-axis) and admixed American populations (y-axis). European reference populations are grouped according to their geographic origins as shown below the plot.

Supplementary Table S1. Geographical origins of mtDNA (maternal) and Y-DNA (paternal) haplotypes for the population of Chocó.

mtDNA (maternal lineage)			
n	mtDNA Haplotype	Geographical Origin	Continental Ancestry
4	A	Americas, Siberia, East Asia	Native America
14	B4'5	Americas, Asia, Polynesia	Native America
1	D	Americas, Asia	Native America
3	L0a	Central, Eastern, Southern Africa	Africa
1	L1b	Coastal Western Africa	Africa
8	L1b1a	Coastal Western Africa	Africa
10	L1c	Central Africa	Africa
1	L2	Sub-Saharan Africa	Africa
15	L2a	Sub-Saharan Africa	Africa
1	L2b	Sub-Saharan Africa	Africa
7	L2c	Sub-Saharan Africa	Africa
1	L2d	Sub-Saharan Africa	Africa
7	L3b	Africa	Africa
5	L3d	Northern, Eastern, Southern Africa	Africa
20	L3e	Africa	Africa
2	L3f	Northern, Eastern, Coastal Western Africa	Africa
Y-DNA (paternal lineage)			
n	Y-DNA Haplotype	Geographical Origin	Continental Ancestry
2	E	Africa, Europe, Near East	Africa
27	E1b1a	Africa	Africa
1	J1	Southern Europe, Near East, Northern Africa	Middle East
1	J2	Southern Europe, Near East, Northern Africa	Middle East
8	R1b1b2	Europe	Europe

Supplementary Table S2. Tree-based f3 test of African ancestry sources of admixed American populations.

	f_3(X; European,Yoruba)[1]	f_3(X; European,Mende)[2]	(Yoruba-Mende)/Mende[3]	Z-diff[4]
Chocó	-0.0252	-0.0248	0.0189	-4.09
Medellín	-0.0053	-0.0053	0.0059	-0.09
African American	-0.0301	-0.0297	0.0154	-5.62
African Caribbean	-0.0185	-0.0178	0.0404	-7.52
Puerto Rico	-0.0167	-0.0167	0.0026	-0.14

[1]The f3 test statistic value between either the Spanish population (for Chocó, Medellín and Puerto Rico) or the British population (for African American and African Caribbean) and the Yoruba population from Nigeria; a lower f3 value indicates a stronger match. [2]The f3 test statistic value between either the Spanish population (for Chocó, Medellín and Puerto Rico) or the British population (for African American and African Caribbean) and the Mende population from Sierra Leone; a lower f3 value indicates a stronger match. [3]The fold increase in magnitude for the Yoruba f3 value over the Mende f3 value; a higher value indicates a stronger match for the Yoruba population. [4]The Yoruba f3 test statistic Z-score minus the Mende f3 test statistic Z-score; a lower difference value indicates a stronger match for the Yoruba population.

GEOGRAPHIC POPULATION STRUCTURE IN CHOCÓ

The existence of geographic population structure in Chocó was interrogated by analyzing the distribution of the donors' parents' geographical origins across genetically defined groups. Donors were asked to identify the geographic origins of their parents, and parental origins were assigned to six regions from Chocó as well as two additional administrative departments (*i.e.*, states) in Colombia: Antioquia (Medellín) and Cauca (Supplementary Figure S2). The program fineSTRUCTURE (Lawson et al. 2012) was used to group the same individuals from Chocó into five major genetic clusters (Supplementary Figure S3). Distributions of paternal and maternal geographic origins, quantified as normalized counts, were then determined for each genetically clustered group of donors (Supplementary Figure S4A). The observed pairwise distances between genetic group-specific geographic origin distributions were computed separately for paternal and maternal origins. Geographic pairwise distances between genetically defined groups were computed as the Euclidean distance (d_{ij}) between vectors of geographic origin fractions for each group:

$$d_{group=i\,\&\,group=j} = \sum_{\forall\, regions} \left(f_{group=i,region=n} - f_{group=j,region=n}\right)^2$$

Distributions of the observed maternal and paternal geographic distances computed in this way were compared to expected distributions computed by randomly permuting individuals, maintaining their parental origin identifications, among the genetically defined groups (Supplementary Figure S4B).

The maternal genetic lineage does not show evidence of population genetic structure, as the observed versus expected pairwise geographic distances are not significantly different. Observed geographic distances based on paternal origin assignments are significantly greater than expected by chance, consistent with geographic population genetic structure for the paternal lineage in Chocó. Nevertheless, the difference seen between observed and expected paternal geographic distances is fairly small and only marginally significant.

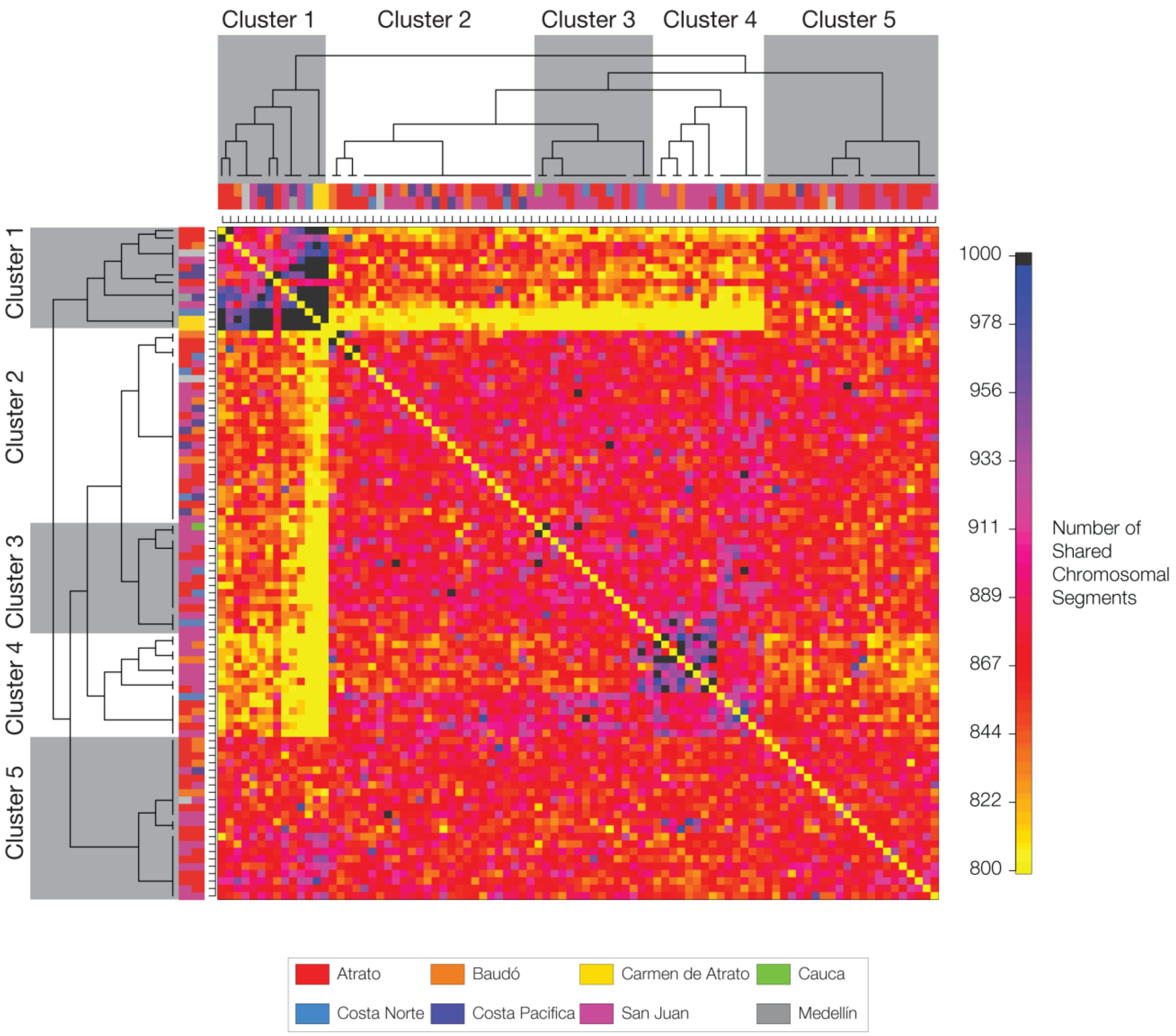

Supplementary Figure S12 Genetic relationships among individuals from Chocó and their parental geographic origins. The program fineSTRUCTURE was used to cluster individuals from Chocó into five major genetic clusters, based on the number of chromosomal segments shared between individuals. For each individual, their maternal and paternal geographic origins are color-coded and placed alongside the dendograms as shown in the key below the plot.

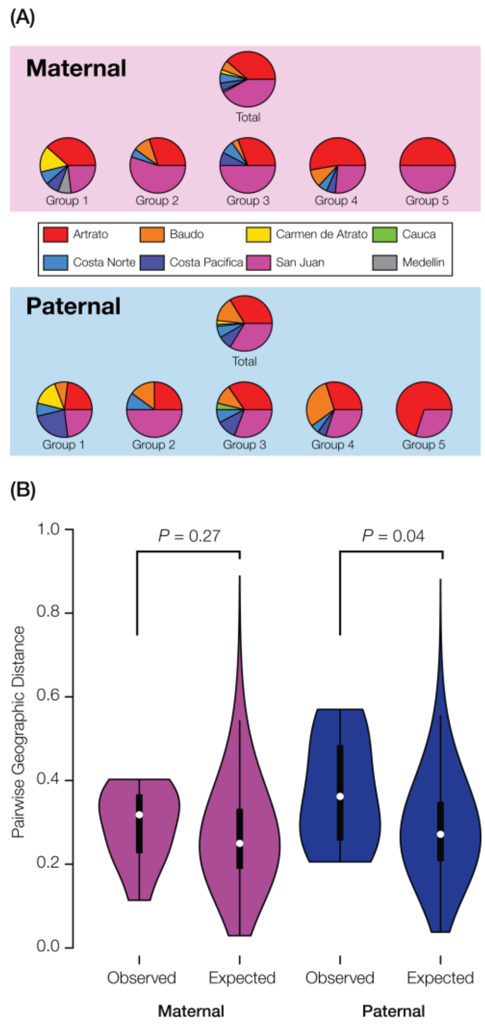

Supplementary Figure S13 Geographical population structure in Chocó. (A) The maternal (top panel) and paternal (bottom panel) parental origins for Chocó sample donors from the five main genetic clusters identified by fineSTRUCTURE (Supplementary Figure S2). (B) Distributions of observed versus expected geographic distances among genetically identified clusters shown for the maternal and paternal lineages; *P*-values show the significance of the difference between the distributions.

POPULATION STRUCTURE AND INTERNALLY DISPLACED PERSONS IN CHOCÓ

Sample donors from the ChocoGen project were chosen in an effort to represent a broad swath of the geographic diversity of the region (Supplementary Figure S2A). Donors' geographic origins were independently assessed for both the maternal and paternal lineages, and geographic diversity along these two lineages was compared for genetically defined groups in order to assess the extent of geographic population structure that exists in Chocó. The population of Chocó shows evidence for geographic structure along the paternal lineage but not for the maternal lineage (Supplementary Figure S13). These results are consistent with prior studies that have uncovered sex-biased patterns of human migration, whereby females show higher levels of migration than males, and accordingly lower levels of geographic population structure (Seielstad et al. 1998). However, the level of geographic population structure we observed for the paternal lineage in Chocó, along with the differences seen between the maternal and paternal lineages, are low and only marginally significant. The relatively low levels of observed population geographic structure could be a result of the large documented presence of internally displaced persons in Chocó as a result of the long running civil war in Colombia. Chocó has disproportionately suffered the effects of this armed conflict; the United Nations High Commissioner for Refugees estimates that Colombia has ~5.8 million internally displaced persons, one-third of whom are located in the Pacific region of the country, including the state of Chocó.
